# Supplementary material for: Predicting the first steps of evolution in randomly assembled communities
Source: Nat Commun. 2024 Oct 1;15:8495. doi: 10.1038/s41467-024-52467-3 (PMC11445446; doi:10.1038/s41467-024-52467-3)
Supplement: Supplementary file 1 — Supplementary Information [file 41467_2024_52467_MOESM1_ESM.pdf]

Predicting the First Steps of Evolution in Randomly Assembled Communities: Supplementary Notes

John McEnany[1] and Benjamin H. Good[2,3,4,*]

[1]*Biophysics Program, Stanford University, Stanford, CA 94305, USA*
[2]*Department of Applied Physics, Stanford University, Stanford, CA 94305, USA*
[3]*Department of Biology, Stanford University, Stanford, CA 94305, USA*
[4]*Chan Zuckerberg Biohub – San Francisco, San Francisco, CA 94158, USA*

*Correspondence should be addressed to: B.H.G. (bhgood@stanford.edu)

Contents

Page

Supplementary Note 1: Model Outline **13**
1.1 General Ecological Dynamics 13
1.2 Coarse-Grained Ecological Dynamics 14
1.3 Incorporating Simple Forms of Cross-feeding 16
1.4 Defining the Local Species Pool 17
1.5 Ecological Equilibrium as Optimization 17
1.6 Analytic Approximation of Ecological Equilibrium 18

Supplementary Note 2: Replica-Theoretic Analysis of First-Step Invasion **20**
2.1 Single-Ecosystem Approximation of First-Step Invasion 20
2.2 Partition Function and the Replica Trick 21
2.3 Introducing First-Step Mutations 24
2.4 Saddle Point Approximations 25
2.5 Joint Distribution of Δ_P and Δ_M 27
2.6 Parent-Mutant Coexistence Probability 30

Supplementary Note 3: Scaling Analysis of First-Step Mutations **31**
3.1 Sampling Depth and Fitness Gauge 31
3.2 Niche Saturation and σ_{inv} 32
3.3 Scaling Analysis of $\mathbb{P}_{\text{coex}}$ 34
3.4 Dependence of $\mathbb{P}_{\text{coex}}$ on Invasion Fitness and Abundance 36
3.5 Self-Consistency of Replica Approximations 37

Supplementary Note 4: Extensions of Model Assumptions **39**

4.1 Non-Uniform Resource Supply 39
4.2 Variation in Number of Metabolized Resources 40
4.3 Metabolic Trade-offs in Sampling 42
4.4 Continuous Resource Usage 42
4.5 Specialist Community Assembly 43

Supplementary Note 5: Simulations and Numerics **44**
5.1 Community Simulations 44
5.2 Theory Numerics 45

List of Supplementary Figures

1 Explicit community dynamics with dilution 3
2 Distribution of fitness effects for a single organism 4
3 Alternative consumer resource models 5
4 Simulations with returning species 6
5 Mutant-parent coexistence for alternate strategy mutations 7
6 Dependence of mutant-parent coexistence on invasion fitness and background abundance 8
7 Distribution of fitness effects and mutant-parent coexistence probabilities under heterogeneous resource supply. 9
8 Fitness effects and mutant-parent coexistence for different types of metabolic trade-offs 10
9 Fitness effects and mutant-parent coexistence with a range of specialist and generalist resource strategies 11
10 Checks for numerical stability 12

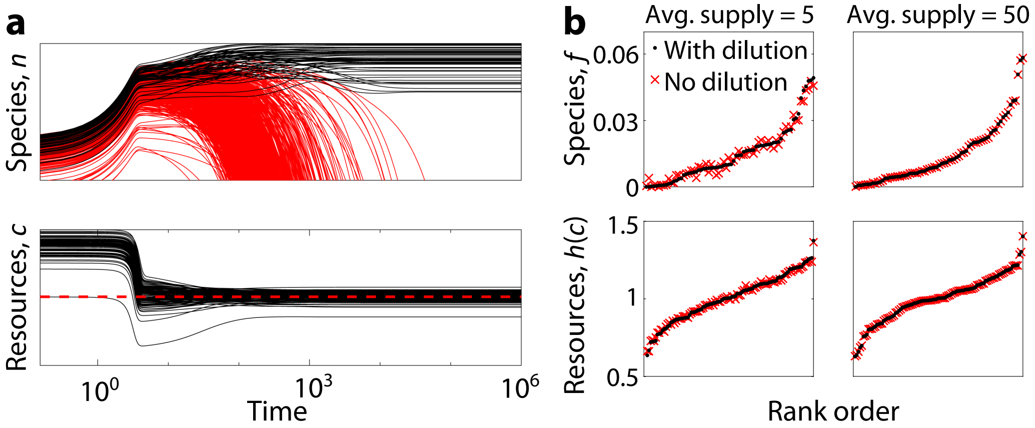

Supplementary Figure 1: Explicit community dynamics with dilution. **(a)** Absolute abundance of species (top) and resource concentrations (bottom) over time on a log-log scale, for an example community simulated by explicitly integrating Eq. (S1a). Red curves are species which go extinct at equilibrium; the red dashed line in the lower plot shows the expected average value of c at equilibrium. **(b)** Equilibrium species relative abundances and resource availabilities $h(c)$, obtained from explicit simulation (black dots) and finding the equilibrium through optimization, ignoring the dilution term (red crosses). Predictions are shown for two values of average resource supply $\overline{K}$. Simulations were performed with $\mathcal{R} = 100$, $\mathcal{R}_0 = 20$, $\mathcal{S}/\mathcal{R} = 10$, $\mathrm{Std}(X_\mu) = 0.5/\mathcal{R}_0$, and $\mathrm{Var}(K)/\overline{K}^2 = 0.1$. The uptake function $u(c)$ was taken as a Monod function, $u_i(c_i) = u_{\max} c_i/(c_{1/2} + c_i)$, with $u_{\max} = 4$ and $c_{1/2} = 1$.

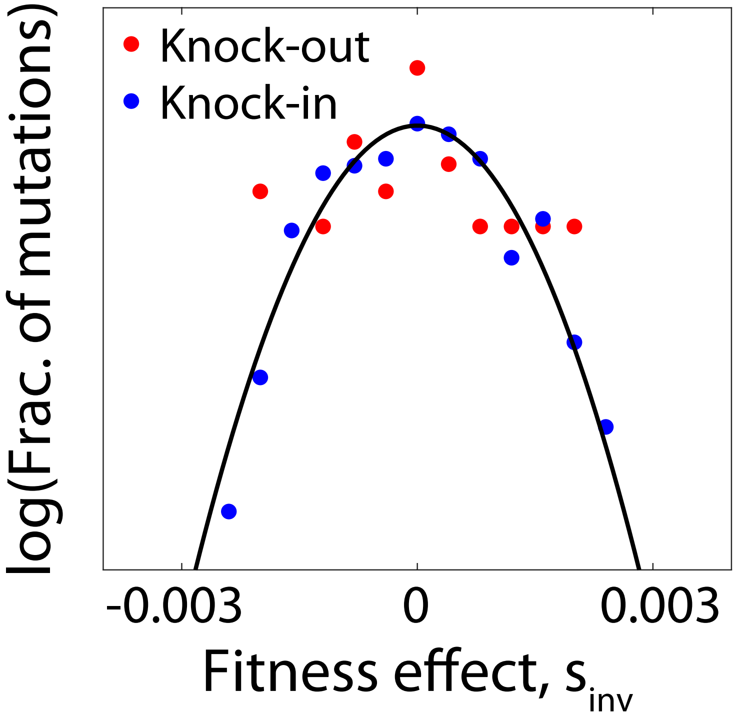

Supplementary Figure 2: Distribution of fitness effects for a single organism. Distribution of invasion fitnesses for knock-out and knock-in strategy mutations of a single organism within a single sampled community. Black curve shows Gaussian theory prediction, while dots are histogram values over all possible strategy mutations. Simulation was run for $\mathcal{S}^*/\mathcal{R} = 0.8, \mathcal{R} = 200$, $\mathcal{R}_0 = 40$, $\mathcal{S}^*/\mathcal{S} = 0.1$, and uniform resource supply.

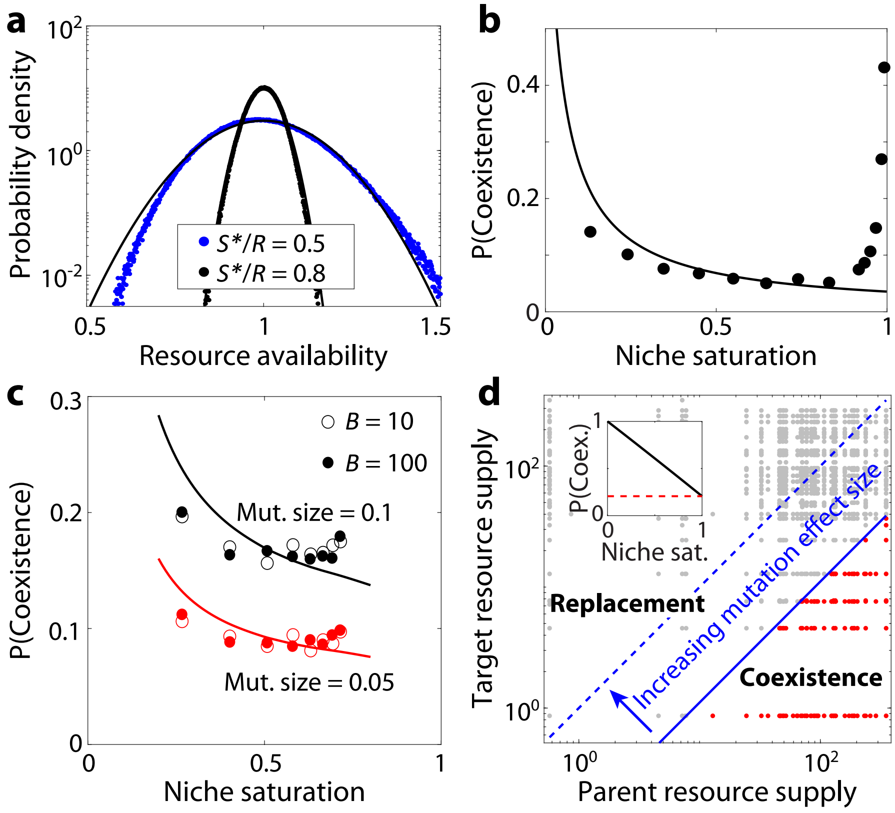

Supplementary Figure 3: Alternative consumer resource models. Our main conclusions about mutation in assembled communities can be generalized to other consumer-resource sampling structures. **(a)** Distribution of resource availabilities h_i for an ecosystem assembled with strategy vectors drawn from the Dirichlet distribution as described in Supplementary Note 4.4. Black curves show theoretical prediction from the binary resource use case studied in this paper, while points show simulated results. Simulations were run with $\mathcal{R} = 200$, $\mathcal{R}_0 = 40$ and $\mathcal{S}^*/\mathcal{S} = 0.1$ for uniform resource supply. **(b)** Probability of mutant-parent coexistence for knock-out strategy mutations in this Dirichlet assembled community, with the same parameters as in (a). Black curve shows the theoretical prediction for binary resource use. **(c)** Mutant-parent coexistence probability for a community where all species consume all resources at similar levels (Supplementary Note 4.4), for different mutation effect sizes Γ. Curves show theoretical prediction from the binary model, with an effective value of $\mathcal{R}_0$ corresponding to the shape parameter B of the Dirichlet distribution. Other parameters were $\mathrm{Std}(X_\mu) = 1/B\mathcal{R}$ and $\mathcal{R} = 200$. **(d)** Conditions for mutant-parent coexistence in a specialist community (Supplementary Note 4.5). Each point corresponds to a beneficial mutation in a 50-member community, with red points showing mutant-parent coexistence. Axes indicate the supply of the resource the parent consumes, and the supply of the "target" resource the mutant gains access to. The blue line is the predicted boundary between coexistence and replacement for mutations with effect size $\gamma_{\mathrm{spec}} = 0.1$. Inset: Mutant-parent coexistence probability as a function of niche saturation; the red dashed line corresponds to a saturated community, as shown in the rest of (d).

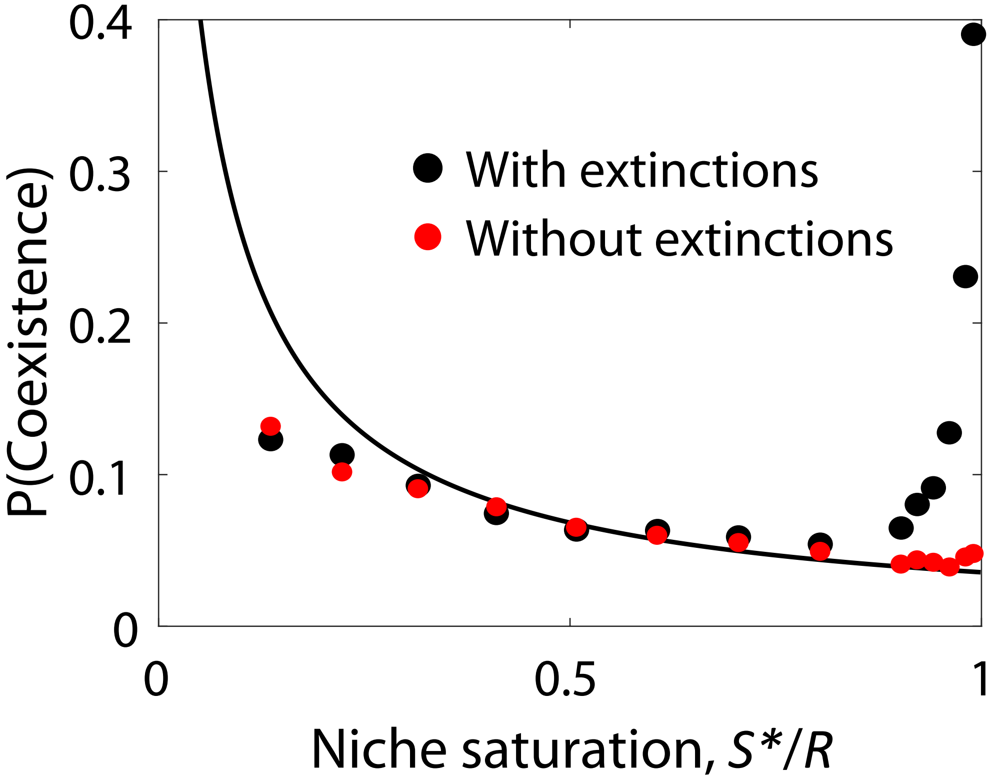

Supplementary Figure 4: Simulations with returning species. Probability of coexistence as a function of niche saturation, with simulations using the "simultaneous assembly" approximation where mutant invasion is modeled using a single assembly process (Supplementary Note 2.1). Red points show the simultaneous assembly approximation, which allows reappearance of extinct species. Black points show simulations where extinct species were disallowed from returning (as in the rest of our study); this change results in coexistence probability increasing at high saturation. Simulations were performed with $\mathcal{R} = 200$, $\mathcal{R}_0 = 40$, and $\mathcal{S}^*/\mathcal{S} = 0.1$ using knock-out mutations.

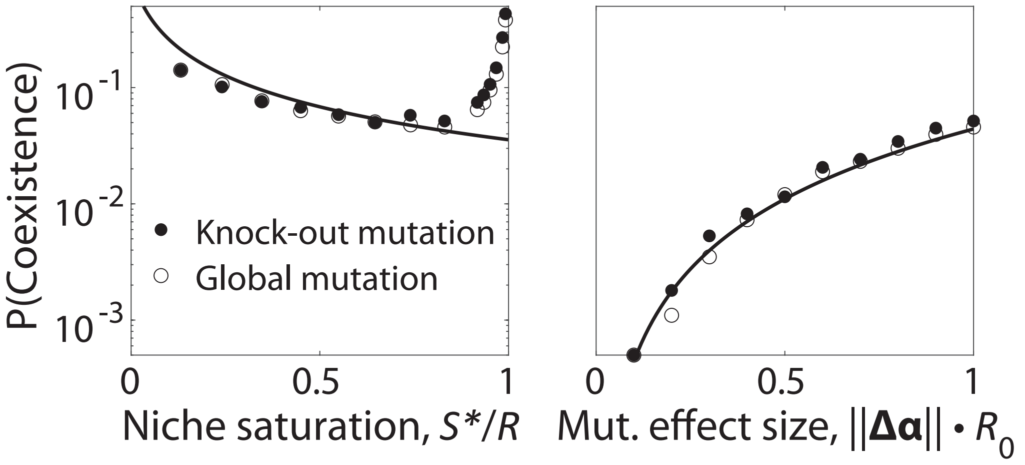

Supplementary Figure 5: Mutant-parent coexistence for alternate strategy mutations. Left: Mutant-parent coexistence probability as a function of niche saturation $\mathcal{S}^*/\mathcal{R}$ for full knock-out mutations and "global" strategy mutations with $\|\Delta\boldsymbol{\alpha}\| = 1/\mathcal{R}_0$ (which have similarly-sized changes to all nonzero entries of $\boldsymbol{\alpha}$, as described in Supplementary Note 4.4). Right: Mutant-parent coexistence probability as a function of mutation effect size $\|\Delta\boldsymbol{\alpha}\|$ for $\mathcal{S}^*/\mathcal{R} = 0.8$, for partial knock-out and global strategy mutations. Black curves show theoretical prediction. All simulations run with $\mathcal{R} = 100$, $\mathcal{R}_0 = 40$, and $\mathcal{S}^*/\mathcal{S} = 0.1$ using Dirichlet-distributed resource usage vectors.

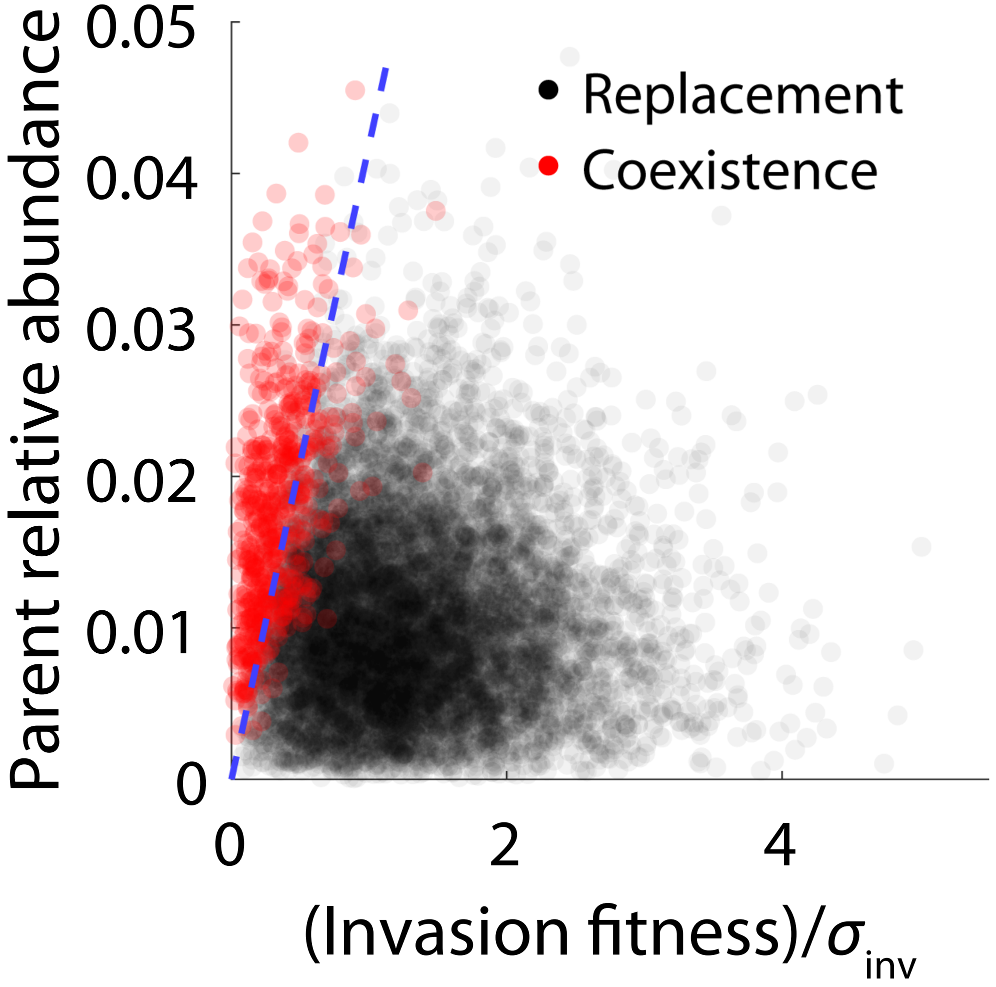

Supplementary Figure 6: Dependence of mutant-parent coexistence on invasion fitness and background abundance. Scatterplot showing the invasion fitness of knock-out strategy mutations against the relative abundance of the parent before mutant invasion, with colors indicating whether the mutants coexist with or replace their parent at ecological equilibrium. The dashed blue line shows the theoretical prediction that should divide replacement events from coexistence events. Simulations run for $\mathcal{R} = 200$, $\mathcal{R}_0 = 40$, $\mathcal{S}^*/\mathcal{S} = 0.1$, and $\mathcal{S}^*/\mathcal{R} = 0.8$.

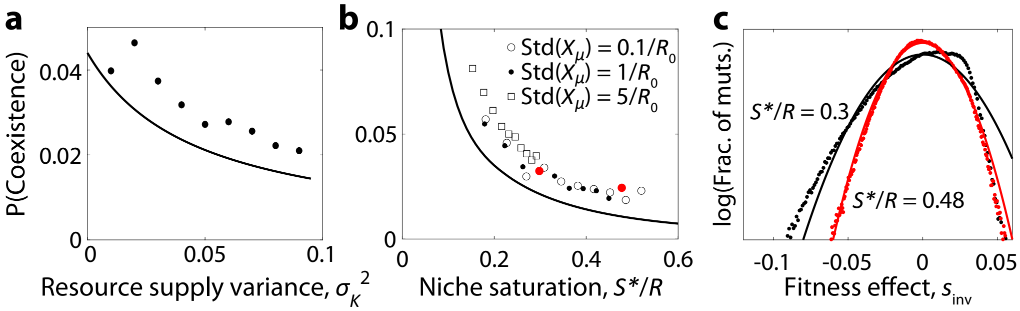

Supplementary Figure 7: Distribution of fitness effects and mutant-parent coexistence probabilities under heterogenous resource supply. **(a)** Mutant-parent coexistence probability for knockout mutants as a function of scaled variance in resource supply $\sigma_K^2 \equiv \mathrm{Var}(K)/\overline{K}^2$, with $\mathcal{R} = 200$, $\mathcal{R}_0 = 40$, $\mathcal{S}^*/\mathcal{S} = 0.1$, and $\mathcal{S}^*/\mathcal{R} = 0.8$. **(b)** Coexistence probability of parents and knock-out mutants for communities assembled with exponentially distributed resource supply (corresponding to $\sigma_K^2 = 1$), and different levels of uptake budget variation $\mathrm{Std}(X_\mu)$ among sampled organisms. Simulations were run with $\mathcal{R} = 150$ and $\mathcal{R}_0 = 30$. **(c)** Distribution of fitness effects for knock-out mutants for communities corresponding to the red points in (b). In all plots, points show simulation results while curves show theory predictions.

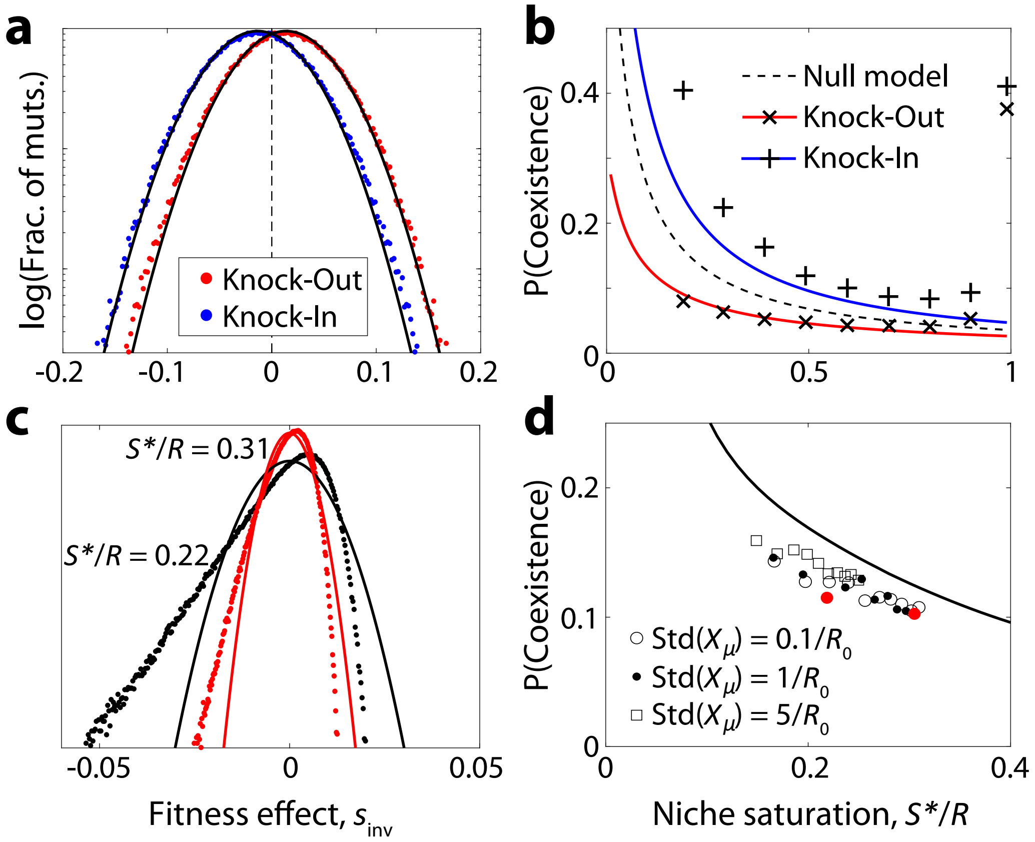

Supplementary Figure 8: Fitness effects and mutant-parent coexistence for different types of metabolic trade-offs. **(a)** Distribution of invasion fitnesses for the alternative model in Ref. (1), where the cost of using a resource contributes to the death rate of an organism, rather than occupying a portion of its overall resource uptake budget. Black curves show theoretical prediction for knock-in and knock-out mutations, while points show simulation results. Simulations were run with $\mathcal{R} = 200$, $\mathcal{R}_0 = 40$, $\mathcal{S}^*/\mathcal{S} = 0.1$, and $\mathcal{S}^*/\mathcal{R} = 0.8$. **(b)** Mutant-parent coexistence probability for the Ref. (1) CRM with the same parameters as (a), as a function of niche saturation, $\mathcal{S}^*/\mathcal{R}$. The average fitness benefit (cost) for knock-out (knock-in) mutations effectively gives strategy mutations a nonzero change in pure fitness, which lowers (raises) the theoretically predicted coexistence probability relative to the neutral case. Red and blue curves show these adjusted predictions, while crosses and pluses show simulated results. **(c)** Distribution of fitness effects for knock-out mutations in a community where sampled species lack metabolic trade-offs, as described in Supplementary Note 4.3. Simulations run with $\mathcal{R} = 150$ and $\mathcal{R}_0 = 30$. **(d)** Mutant-parent coexistence probability for the same ecosystem as a function of niche saturation, for different levels of variation in uptake budget $\mathrm{Std}(X_\mu)$. Red points correspond to the parameter values in (c).

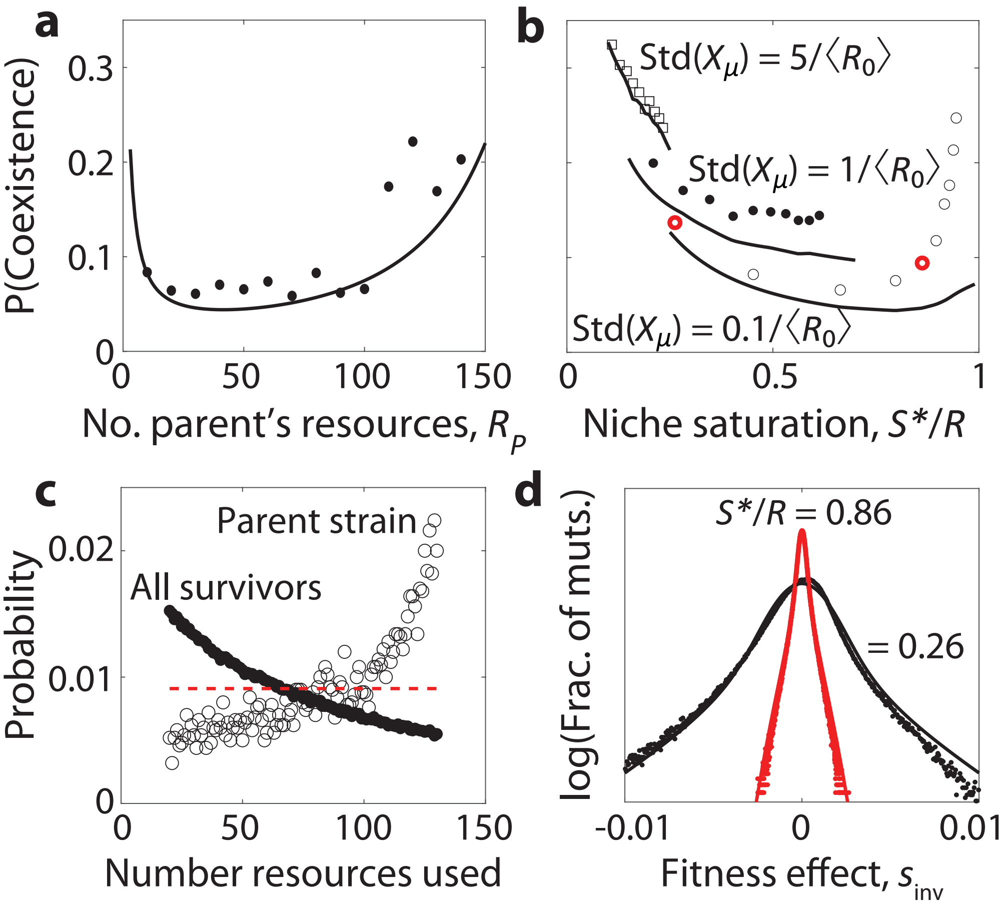

Supplementary Figure 9: Fitness effects and mutant-parent coexistence with a range of specialist and generalist resource strategies. **(a)** Probability that the mutant coexists with its parent strain as a function of the number of resources $\mathcal{R}_P$ used by the parent, in a background community with $\mathcal{R}_0 = 40$. Points denote simulation results for $\mathcal{R} = 200$, $\mathcal{S}^*/\mathcal{S} = 0.1$, and $\mathcal{S}^*/\mathcal{R} = 0.8$. **(b)** Mutant-parent coexistence probability when the number of resources used by each strain is uniformly distributed between 30 and 120 (Supplementary Note 4.2). Points denote simulation results for knock-out mutations with $\mathcal{R} = 150$, and different levels of variation in uptake budget $\mathrm{Std}(X_\mu)$. **(c)** Distribution of the actual number of resources used by surviving species in the community corresponding to the rightmost red circle in (b), including all survivors (closed circles) and the subset of species which produced a successful knock-out mutation (open circles). The red dashed line shows the corresponding uniform distribution for sampled species. **(d)** Distribution of knock-out fitness effects for the community in (b), with parameter values corresponding to the red circles. All points show simulation results, while curves show theory predictions, using the distribution in (c) as an input.

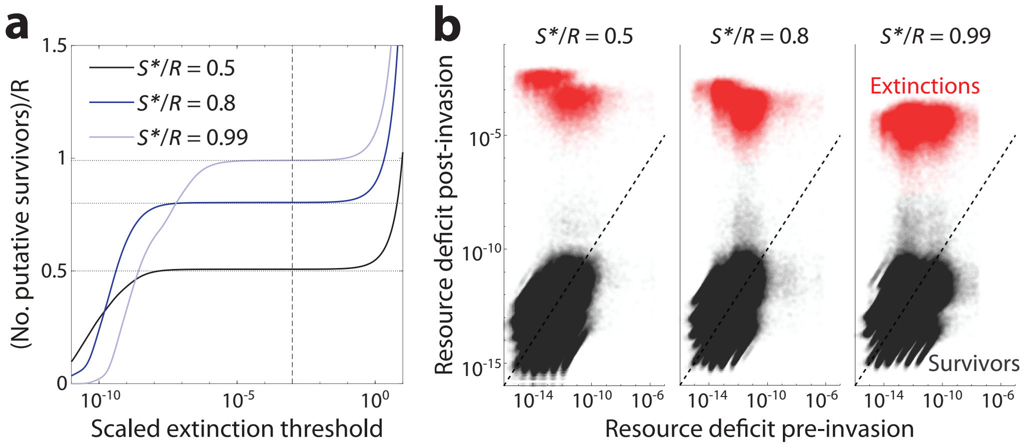

Supplementary Figure 10: Checks for numerical stability. (a) Plot of the number of putatively identified survivors for an assembled community, as a function of the numerical extinction threshold. If the resource deficit for an organism is above this threshold times $\mathrm{Std}(h_i)$, the organism is labeled extinct. The plateau over multiple orders of magnitude indicates a numerically stable definition of survival vs. extinction; the dashed line shows the threshold used throughout our analysis. **(b)** Scatterplot of numerical resource deficit of organisms before and after invasion of a knock-out strategy mutant, with color indicating whether the resource deficit after invasion passes the extinction threshold, (i.e., whether the species has gone extinct). The fact that most red and gray points are clearly separated shows that most species identified as extinct are insensitive to our precise choice of threshold. Simulations were run with $\mathcal{R} = 200$, $\mathcal{R}_0 = 40$, $\mathcal{S}^*/\mathcal{S} = 0.1$.

Supplementary Note 1: Model Outline

In this section, we outline the setup that allows us to derive our main theoretical results. First, we describe the consumer resource model we study, and how its dynamics can be coarse-grained to a limit where species consume most of the available resources. The task of finding an ecological equilibrium in this coarse-grained model can be described as an optimization problem. Then, we define a convenient fitness gauge which brings our model in line with previous work.

1.1 General Ecological Dynamics

As summarized in the Model section, we are interested in the following system: consider an ecosystem formed by combining $\mathcal{S}$ species, identically and independently drawn from some underlying distribution, competing for $\mathcal{R}$ resources. We allow this ecosystem to come to ecological equilibrium, at which point $\mathcal{S}^* \leq \mathcal{S}$ species will survive at nonzero abundance. Then, suppose that one randomly selected surviving species undergoes a mutation to produce a new strain, which can differ from its parent in its ability to consume resources. If the mutant has positive invasion fitness, it will rise to nonzero abundance in the population, and the abundances of other species may change as well. We are interested in analyzing the properties of this new ecosystem with a single mutant, particularly the probability that the mutant and its parent strain are able to coexist at ecological equilibrium.

First, we define our community assembly process and consumer resource model, which generally follows previous work (1–7). We consider a well-mixed community formed from the combination of $\mathcal{S}$ strains, indexed by $\mu \in \{1, ..., \mathcal{S}\}$, and $\mathcal{R}$ resources, indexed by $i \in \{1, ..., \mathcal{R}\}$. Each organism is associated with a resource uptake vector, $\mathbf{r}_\mu = (r_{\mu,1}, \ldots, r_{\mu,\mathcal{R}})$, which could represent the expression of enzymes used to metabolize each resource. The consumer-resource dynamics for the strain abundances (n_μ) and the local resource concentrations (c_i) are given by Eq. (1) in the main text:

$$\frac{\partial n_\mu}{\partial t} = \sum_{i=1}^{\mathcal{R}} Y_{\mu,i} u_i(\mathbf{c}) r_{\mu,i} n_\mu - \delta \cdot n_\mu \tag{S1a}$$

$$\frac{\partial c_i}{\partial t} = K_i - \sum_{\mu=1}^{\mathcal{S}} u_i(\mathbf{c}) r_{\mu,i} n_\mu - \delta \cdot c_i, \tag{S1b}$$

where δ is the dilution rate, K_i is the input flux of each resource, $u_i(\mathbf{c}) r_{\mu,i} n_\mu$ is the total uptake rate of resource i by strain μ, and $Y_{\mu i}$ is the yield of resource i when converted into biomass by strain μ. For simplicity, we will assume that this yield matrix can be factored into a rank-one form $Y_{\mu,i} = a_i / b_\mu$. While this assumption can approximately account for effects like different stoichiometric yields and cell sizes, it neglects idiosyncratic effects such as resource sequestration (8), where some organisms remove resources from the environment without corresponding growth in abundance. This simplification allows us to choose "biomass-equivalent" units such that $Y_{\mu,i} = 1$, yielding a conservation of mass equation

corresponding to a symmetry between resource consumption and biomass growth:

$$\frac{\partial}{\partial t}\left[\sum_i c_i + \sum_\mu n_\mu\right] = \sum_i K_i - \delta\left[\sum_i c_i + \sum_\mu n_\mu\right] . \tag{S2}$$

The total mass rapidly approaches the $\mathcal{S} + \mathcal{R} - 1$ dimensional manifold,

$$\sum_i c_i(t) + \sum_\mu n_\mu(t) = \frac{\sum_i K_i}{\delta} , \tag{S3}$$

on a timescale proportional to the inverse dilution rate δ^{-1} (which is equivalent to the average generation time at steady state). Since we are interested in the dynamics on timescales that are much longer than a single generation, we assume that Eq. (S3) always applies.

1.2 Coarse-Grained Ecological Dynamics

The dynamics in Eq. (S1) can be further simplified if we assume that the resource uptake rates are sufficiently fast, and the total biomass of organisms sufficiently large, that most supplied resources are consumed by organisms rather than diluted out. When this is the case, we can coarse-grain over time such that the resource concentrations are instantaneously set by the abundance of each strain, rather than being independent dynamical variables ("integrating out the resources"). While this procedure follows previous work (4, 6), we will restate this approximation and its self-consistency conditions for completeness.

As shown by Equation (S1), it is reasonable to approximate that most resources are converted to biomass rather than diluted out when the local concentration of each resource is much smaller than $c_i^{\max} \equiv K_i/\delta$, which is the equilibrium value that would be obtained in the absence of any microbes. In other words, the community must be able to deplete each resource far below the level that would be expected from the environmental supply rates alone. When this condition is satisfied, the total biomass in Eq. (S3) will be dominated by the microbial contribution,

$$\sum_\mu n_\mu(t) \approx \frac{\sum_i K_i}{\delta} , \tag{S4}$$

and the uptake rates of each resource will approach a local steady state set by the current strain abundances:

$$u_i(\mathbf{c}) \approx \frac{K_i}{\sum_\mu n_\mu r_{\mu,i}} \approx \left(\frac{K_i}{\sum_j K_j}\right) \frac{\delta}{\sum_\mu f_\mu r_{\mu,i}} , \tag{S5}$$

where we have defined the relative abundance variables,

$$f_\mu \equiv \frac{n_\mu}{\sum_\nu n_\nu} \approx \frac{\delta \cdot n_\mu}{\sum_i K_i} . \tag{S6}$$

Substituting these expressions into Eq. (S1a) yields the coarse-grained model in Eq. (2) in the Main Text:

$$\frac{\partial f_\mu}{\partial \tau} = f_\mu \left[\sum_{i=1}^{\mathcal{R}} r_{\mu,i} h_i(\mathbf{f}) - 1 \right] , \tag{S7a}$$

where time is measured in generations ($\tau \equiv \delta \cdot t$) and

$$h_i(\mathbf{f}) \equiv \frac{u_i(\mathbf{c})}{\delta} \approx \left(\frac{K_i}{\sum_j K_j} \right) \left[\sum_\mu r_{\mu,i} f_\mu \right]^{-1} \tag{S7b}$$

can be interpreted as the local availability of resource i.

Self-consistency of coarse-grained dynamics. These coarse-grained dynamics will be self-consistent when the relative abundances that are attained in Eq. (S7) satisfy

$$u_i^{-1} \left(\frac{K_i}{\sum_j K_j} \frac{\delta}{\sum_\mu r_{\mu,i} f_\mu} \right) \ll \frac{K_i}{\delta} , \tag{S8}$$

where $u_i^{-1}(\cdot)$ is the inverse of the uptake function $u_i(\mathbf{c})$. Since the relative abundances only depend on the relative supply rates, $K_i/\sum_j K_j$, this condition can always be satisfied if δ is sufficiently small, or if the total biomass is sufficiently large. For example, if the uptake rates can be described by a Monod function,

$$u_i(\mathbf{c}) = \frac{u_{\max,i} \cdot c_i}{c_i^* + c_i} , \tag{S9}$$

then the condition in Eq. (S8) reduces to

$$\frac{K_i}{c_i^* \delta} \left[\frac{u_{\max,i}}{\delta} \frac{\sum_j K_j}{K_i} \sum_\mu r_{\mu,i} f_\mu - 1 \right] \gg 1 . \tag{S10}$$

This will be valid for small enough values of δ, or large enough values of $\sum_j K_j / K_i$.

Rescaled variables. In our analysis below, it will be convenient to reparameterize the model in Eq. (S7) by decomposing the resource uptake vector $\mathbf{r}_\mu$ into an overall "uptake budget" $X_\mu \equiv \log \sum_i r_{\mu,i}$, and a normalized "strategy vector" $\boldsymbol{\alpha}_\mu \equiv \mathbf{r}_\mu / \sum_i r_{\mu,i}$, which contains $\mathcal{R}$ non-negative entries that sum to one. The original uptake rates can then be expressed as

$$r_{\mu,i} = \alpha_{\mu,i} e^{X_\mu} . \tag{S11}$$

The uptake budget dictates the overall capacity of the organism to consume resources, setting its maximum growth rate under ideal growth conditions; the strategy vector describes how the organism allocates this uptake budget between the different substitutable resources. This alternative parametrization is equivalent to the original basis $\mathbf{r}_\mu$, but will lead to simpler expressions in many of our analytical calculations below.

To streamline notation, it will also be useful to define the relative resource supply rates,

$$\kappa_i \equiv \frac{K_i}{\frac{1}{\mathcal{R}}\sum_j K_j}\,, \tag{S12}$$

which describe how the supply of a given resource deviates from the average value, $\overline{K} = \frac{1}{\mathcal{R}}\sum_i K_i$. The relative supply rates are normalized such that $\frac{1}{\mathcal{R}}\sum_i \kappa_i = 1$. We will also refer to the variance of κ_i, $\sigma_K^2 \equiv \mathrm{Var}(\kappa) = \mathrm{Var}(K)/\overline{K}^2$.

1.3 Incorporating Simple Forms of Cross-feeding

While the derivation of Eq. (S7) assumed that the resources were externally supplied, similar dynamics can continue to hold for simplified forms of cross-feeding, provided that κ_i is replaced by an effective supply rate that accounts for internal metabolic conversion. To see this, we consider the "universal" cross-feeding model from Ref. (9), where the metabolism of each resource is associated with a corresponding "leakage rate" ℓ_i, which describes the proportion of that resource that is excreted back into the environment in the form of other resources, rather than being converted into biomass. This conversion process is described by a stoichiometric matrix $\mathbf{D}$, whose entries $D_{i,j}$ represent the fraction of resource j that is excreted in the form of resource i. This model assumes that every organism capable of metabolizing a resource produces the same byproducts in the same proportions; species can still differ in their overall excretion profiles, but these are completely determined by the corresponding uptake rates $\mathbf{r}_\mu$. These cross-feeding dynamics can be described by a generalization of Eq. (S1),

$$\frac{\partial n_\mu}{\partial t} = n_\mu\left[\sum_{i=1}^{\mathcal{R}} \alpha_{\mu,i} e^{X_\mu}(1-\ell_i)h_i(\mathbf{c}) - 1\right], \tag{S13a}$$

$$\frac{\partial c_i}{\partial t} = K_i - \delta_i c_i + \sum_{\mu=1}^{\mathcal{S}} n_\mu e^{X_\mu}\left[-h_i(\mathbf{c})\alpha_{\mu,i} + \sum_{j=1}^{R} \ell_j D_{i,j} h_j(\mathbf{c})\alpha_{\mu,j}\right], \tag{S13b}$$

where the first term in the brackets of Eq. (S13b) represents the consumption of that resource, while the second term represents the production via metabolism of other resources. If we can continue to make the approximation that the dilution of resources is negligible compared to their consumption by other organisms, then the steady-state resource availabilities $h_i(\mathbf{c})$ will obey a generalization of Eq. (S5),

$$h_i \sum_\mu n_\mu e^{X_\mu}\alpha_{\mu,i} = \sum_j (\mathbf{A}^{-1})_{i,j} K_j, \tag{S14}$$

where $\mathbf{A}$ is an $\mathcal{R}$-by-$\mathcal{R}$ matrix whose entries are given by $A_{i,j} = \delta_{i,j} - \ell_j D_{i,j}$ (note that $\delta_{i,j}$ is the Kronecker delta). This shows that we can recover the same dynamics in Eq. (S7) by defining the effective supply rates,

$$\kappa_i^{\mathrm{eff}} = (1-\ell_i)\sum_j (\mathbf{A}^{-1})_{i,j}\kappa_j = \frac{\sum_j (1-\ell_i)(\mathbf{A}^{-1})_{i,j}K_j}{\sum_{k,\ell}(1-\ell_k)(\mathbf{A}^{-1})_{k,\ell}K_\ell/\mathcal{R}}. \tag{S15}$$

which depends on the external supply rates and the stoichiometry of metabolic conversions, but is otherwise independent of the species that are present in the community.

1.4 Defining the Local Species Pool

All that remains is to choose the resource consumption parameters of the $\mathcal{S}$ initial colonizers. As described in the text, we assume that the phenotypes of each species are drawn from a common statistical distribution. For our theoretical analysis, we follow the procedure of Ref. (1), where the uptake budgets X_μ are drawn from a normal distribution with standard deviation $\epsilon/\mathcal{R}_0$, and the resource consumption strategies $\boldsymbol{\alpha}_\mu$ are normalized binary vectors where each entry has an independent probability $\mathcal{R}_0/\mathcal{R}$ of being nonzero. The independence of X_μ and $\boldsymbol{\alpha}_\mu$ results in a soft metabolic tradeoff, where strains that consume more resources are usually less effective at using any individual one. To test the importance of this and other assumptions, we also perform simulations for several alternative distributions (such as Dirichlet-distributed resource consumption strategies) to verify that our qualitative results are robust (Supplementary Note 4; Supplementary Figures 3, 8, and 9).

1.5 Ecological Equilibrium as Optimization

For any fixed set of species, the ecological dynamics in Eq. (S7) can be shown to possess a convex Lyapunov function,

$$\Lambda(\mathbf{f}) \equiv \frac{1}{\mathcal{R}} \sum_i \kappa_i \log h_i(\mathbf{f}) - \sum_\mu f_\mu \,, \tag{S16}$$

and a single stable equilibrium (see, e.g. Refs. 4, 6, 7). At this equilibrium, none of the surviving strains can increase in abundance, while none of the extinct strains could invade the ecosystem if they were re-introduced at low abundance. Thus, at ecological equilibrium, the bracketed term in Eq. (S7) (the "resource surplus") must be non-positive for all μ, and is equal to zero if and only if species μ survives at nonzero abundance.

Conveniently, the task of finding the unique stable equilibrium in this model can be recast as a constrained optimization problem (1, 4, 10). Specifically, we treat the resource availabilities h_i (rather than the relative abundances f_μ) as free variables to optimize over, and consider the task

$$\begin{aligned} &\text{maximize} \sum_i \kappa_i \log h_i \\ &\text{subject to} \sum_i \alpha_{\mu,i} h_i - e^{-X_\mu} \le 0 \;\forall \mu. \end{aligned} \tag{S17}$$

The constraint is the same as that discussed above, requiring that the resource surplus be non-positive for each organism. This model possesses an important symmetry which will be useful when making approximations later: it is invariant under a rescaling of the total uptake budget, which we can view as a change in "pure fitness" decoupled from the

environment. In particular, if we change variables into translated fitness $\tilde{X}_\mu = X_\mu + \chi$, and rescaled $\tilde{h}_i = e^{-\chi} h_i$, then the optimization problem becomes

$$\begin{aligned} &\text{maximize} \sum_i \kappa_i \left(\log \tilde{h}_i + \chi\right) \\ &\text{subject to } e^{\chi}\left(\sum_i \tilde{h}_i \alpha_{\mu i} - e^{-\tilde{X}_\mu}\right) \leq 0 \;\forall \mu. \end{aligned} \tag{S18}$$

Since χ is just an arbitrary constant, it is clear that these optimization problems are the same, up to the rescaling of the h_i. Suppose that h_i^* solves the first optimization problem. Then, $\tilde{h}_i^* = e^{-\chi} h_i^*$ will solve the gauge-shifted optimization problem

$$\begin{aligned} &\text{maximize} \sum_i \kappa_i \log \tilde{h}_i \\ &\text{subject to} \sum_i \tilde{h}_i \alpha_{\mu i} - e^{-\tilde{X}_\mu} \leq 0 \;\forall \mu. \end{aligned} \tag{S19}$$

To convince ourselves that these two problems really have the same solution, we return to the equation defining h_i in terms of the relative equilibrium abundances f_μ:

$$h_i = \frac{\kappa_i / \mathcal{R}}{\sum_i \alpha_{\mu i} e^{X_\mu} f_\mu}. \tag{S20}$$

Translating the uptake budgets by χ rescales h_i by $e^{-\chi}$, while leaving f_μ unchanged. Therefore, both optimization problems correspond to the same f_μ, which is the actual observable we are interested in. In our numerical simulations, we solve this optimization problem directly, with an arbitrary fitness gauge chosen for numerical convenience.

Once the h_i have been determined, the strain relative abundances f_μ can be calculated numerically. First, the living strains with $f_\mu > 0$ are identified as the strains for which the inequality constraint in Eq. (S17) achieves equality. Given these strains, the definition of h_i in Eq. (S7b) can be inverted to obtain a system of linear equations for f_μ:

$$\sum_\mu r_{\mu,i} f_\mu = \frac{\kappa_i}{\mathcal{R} h_i}. \tag{S21}$$

Because f_μ for extinct strains must be zero, there are only $\mathcal{S}^* \leq \mathcal{R}$ remaining abundances to be solved for. A non-negative linear least squares solver can be used to find the unique set of f_μ that satisfy all $\mathcal{R}$ equations.

1.6 Analytic Approximation of Ecological Equilibrium

By using this gauge symmetry and a small number of additional approximations, we can rewrite this optimization problem in a more analytically tractable form. Indeed, we find that we can put our model in a similar form to that analyzed in Refs. (1) and (10), by

choosing a gauge such that $\langle h_i \rangle = 1$. Let's define a constant h_0 based on the equilibrium solution in our (arbitrary) original gauge,

$$h_0 = \frac{1}{\mathcal{R}} \sum_i h_i^*, \tag{S22}$$

and consider what happens when we set $\chi = \log h_0$. The rescaled solution is

$$\tilde{h}_i^* = \frac{h_i^*}{h_0}, \tag{S23}$$

which by definition has mean 1. It is now natural to define a new variable, $g_i = 1 - \tilde{h}_i$, which has mean zero. The optimization problem is now

$$\begin{aligned} &\text{optimize } \sum_i \kappa_i \log(1 - g_i) \\ &\text{subject to } 1 - e^{-\tilde{X}_\mu} - \sum_i g_i \alpha_{\mu i} \leq 0 \; \forall \mu. \end{aligned} \tag{S24}$$

In large ecosystems, the g_i's will often be small compared to one, allowing us to Taylor expand the optimization function to second order. (We will verify the range of parameters where this holds in Supplementary Note 3.5.) When $g_i \lesssim 1$, the third term on the LHS of the constraint equation must also be less than one. This implies that for any surviving species (where the constraint equation is exactly satisfied) the fitness term $1 - e^{-\tilde{X}_\mu}$ must also be small compared to one. In other words, choosing a gauge where $\langle h_i \rangle = 1$ is the same as choosing a gauge where the pure fitnesses of surviving organisms are centered at zero, allowing us to Taylor expand the $\tilde{X}_\mu$ as well. This relationship arises from the fact that strains with very large differences in total uptake budget are unlikely to coexist. Altogether, the approximate optimization problem is

$$\begin{aligned} &\text{optimize } \sum_i \kappa_i \left(g_i + \frac{g_i^2}{2} \right) \\ &\text{subject to } \tilde{X}_\mu - \sum_i g_i \alpha_{\mu i} \leq 0 \; \forall \mu. \end{aligned} \tag{S25}$$

For organisms that go extinct, the Taylor expansion of the $e^{-\tilde{X}_\mu}$ terms may not be very accurate, since $\tilde{X}_\mu$ can be arbitrarily negative. However, since $1 - \tilde{X}_\mu \leq e^{-\tilde{X}_\mu}$, this approximation will not cause us to erroneously label any extinct species as alive – and for the species which are alive, we will check that the approximation is sufficiently accurate to avoid worry. While it is not yet clear why this approximation is useful, we will see below that remapping the consumer resource problem in this way makes it amenable to replica-theoretic analysis. Note that for numerical simulations, we do not make these approximations, instead solving the original optimization problem in Eq. (S17) – as such, our simulation results implicitly test these assumptions across a range of parameters. We discuss the regime of validity of these assumptions and their interpretations in Supplementary Note 3.5.

Supp. Note 2: Replica-Theoretic Analysis of First-Step Invasion

2.1 Single-Ecosystem Approximation of First-Step Invasion

Having defined our species pool and consumer resource model, we now consider adding first-step mutations into this community. As discussed in the Model section, mutant-parent coexistence can be described as two correlated community assembly processes. Here, we argue that we can reasonably approximate the conditions for mutant-parent coexistence while considering only one community assembly process, which simplifies our theoretical work. First, we describe the two-ecosystem framework. An ecosystem E_1 is formed through standard community assembly: $\mathcal{S}$ species are placed into competition, and $\mathcal{S}^*$ remain alive at equilibrium. Then, one of the surviving species produces a beneficial mutant offspring. This effectively creates a new community assembly problem for ecosystem E_2, where the initial $\mathcal{S}^*$ species plus one mutant are allowed to reach ecological equilibrium. The mutant-parent coexistence probability can therefore be written as

$$\mathbb{P}_{\text{coex}} = \mathbb{P}\left[\text{parent alive in } E_2 | \text{mutant alive in } E_2, \text{parent alive in } E_1\right], \tag{S26}$$

where the conditional probability requires that (1) the mutant be beneficial and (2) the parent be alive when it produces a mutant. Standard manipulation of probabilities allows us to rewrite this as

$$\mathbb{P}_{\text{coex}} = \frac{\mathbb{P}\left[\text{P and M in } E_2\right] - \mathbb{P}\left[\text{P and M in } E_2,\ \text{P not in } E_1\right]}{\mathbb{P}\left[\text{M in } E_2\right] - \mathbb{P}\left[\text{M in } E_2,\ \text{P not in } E_1\right]}. \tag{S27}$$

First, we argue that $\mathbb{P}\left[\text{P and M in } E_2,\ \text{P not in } E_1\right] \approx 0$. This probability describes a situation where the parent is unable to survive in the initial ecosystem, but its hypothetical mutant offspring is. Moreover, the invasion of the mutant offspring adjusts the ecosystem in a way where the original parent strain is also able to simultaneously invade. Intuitively, both of these requirements are unlikely: it will be difficult for a small mutation to allow an otherwise unfavored species to invade, and we would naively expect that the presence of the mutant would make it more difficult for the parent species to survive – not easier – due to their shared competition for resources. Making this approximation, we find

$$\mathbb{P}_{\text{coex}} \approx \frac{\mathbb{P}\left[\text{P and M in } E_2\right]}{\mathbb{P}\left[\text{M in } E_2\right] - \mathbb{P}\left[\text{M in } E_2,\ \text{P not in } E_1 \text{ or } \text{E}_2\right]}. \tag{S28}$$

The next approximation we make is similar: we assume that if the mutant is able to survive, the parent would likely be able to survive in its absence. Mathematically, $\mathbb{P}\left[\text{M in } E_2\right] \gg \mathbb{P}\left[\text{M in } E_2,\ \text{P not in } E_1 \text{ or } \text{E}_2\right]$. While it is possible that a small-effect beneficial mutation could push an organism over the survival threshold, we expect that *most* randomly chosen surviving organisms will not be able to be rescued from extinction by a single mutation. Thus, we write

$$\mathbb{P}_{\text{coex}} \approx \frac{\mathbb{P}\left[\text{P and M in } E_2\right]}{\mathbb{P}\left[\text{M in } E_2\right]}. \tag{S29}$$

Now, our coexistence probability depends only on one ecosystem. But there is still an issue: E_2 is not a traditionally assembled community, since it is formed only from the survivors of

an earlier community assembly process plus a mutant, rather than independently sampled organisms. Let's instead consider E_s, an ecosystem obtained from simultaneous community assembly of $\mathcal{S}$ independently sampled species, plus one "mutant" species which is closely related to one of the $\mathcal{S}$ other species. The key difference between E_2 and E_s is that E_s can potentially contain species which were extinct in E_1, but are brought "back to life" by the invasion of the mutant. While such returns are possible (10), we conjecture that the invasion of a closely-related mutant strain should be a relatively small perturbation to the ecosystem, and as such these re-introductions should be rare – particularly when the number of resources and species are large. So, as a first approximation, we do not expect them to meaningfully impact $\mathbb{P}_{\text{coex}}$. After these approximations, we now have

$$\mathbb{P}_{\text{coex}} \approx \frac{\mathbb{P}\left[\text{P and M in } E_s\right]}{\mathbb{P}\left[\text{M in } E_s\right]}. \tag{S30}$$

This approximation allows us to estimate the mutant-parent coexistence probability in an assembled community by only considering one community assembly process, which includes two closely correlated organisms. (Which one we have labeled the "parent" and "mutant" is essentially arbitrary after making these approximations.) As shown in Supplementary Fig. 4, simulations confirm that these approximations are valid across most of the parameter regime we study, so our one-ecosystem approximation produces results which match the two-ecosystem one. An exception is at very high expected niche saturation, where species "coming back to life" after mutant invasion becomes more relevant. However, there is a narrow range of parameters where this effect is significant (particularly for ecosystems with many resources), and our approximation where these species are allowed to return nonetheless sets a lower bound on the mutant-parent coexistence probability.

2.2 Partition Function and the Replica Trick

We would now like to argue that our model can be treated similarly to Refs. (1) and (10), meaning that their results describe a baseline assembled community before we add mutations. While we will not reproduce every element of their calculation, we restate key steps and approximations. Since ecological equilibrium can be described as the solution to an optimization problem, we can write a partition function for a system with energy $F(\mathbf{g}) = \sum_i F_i(g_i) = \sum_i \kappa_i \left(g_i + \frac{g_i^2}{2}\right)$ at temperature β^{-1}. In the zero-temperature limit $\beta \to \infty$, the system will be exactly at ecological equilibrium; at finite values of β, fluctuations from equilibrium correspond to shot noise in the population. The partition function is

$$Z = \int_{\Omega} \prod_i dg_i e^{-\beta F(\mathbf{g})}, \tag{S31}$$

where Ω corresponds to the constrained region in our optimization problem, where resource surplus is non-positive. We can write out this region explicitly using the Heaviside

function $\theta(x) = \max(0, x)$ as follows:

$$Z = \int_{-\infty}^{1} \prod_i dg_i e^{-\beta F_i(g_i)} \prod_{\mu=1}^{S} \theta\left(\sum_i g_i \alpha_{\mu i} - \tilde{X}_\mu\right) \tag{S32}$$

$$= \int_{-\infty}^{1} \prod_i dg_i e^{-\beta F_i(g_i)} \prod_{\mu=1}^{S} \int d\Delta_\mu \theta(-\Delta_\mu) \delta\left(\Delta_\mu + \sum_i g_i \alpha_{\mu i} - \tilde{X}_\mu\right) \tag{S33}$$

$$= \int_{-\infty}^{1} \prod_i dg_i e^{-\beta F_i(g_i)} \prod_\mu \int \frac{d\Delta_\mu d\hat{\Delta}_\mu}{2\pi} \theta(-\Delta_\mu) e^{i \sum_\mu \hat{\Delta}_\mu \left(\Delta_\mu + \sum_i g_i \alpha_{\mu i} - \tilde{X}_\mu\right)}. \tag{S34}$$

where $\tilde{X}_\mu$ is the pure fitness of species μ, in the gauge described in 6.1.2. We have traded our complicated integration region for two additional integration variables for each species: the resource surplus Δ_μ and its auxiliary Fourier variable $\hat{\Delta}_\mu$.

This partition function is written for a particular choice of $\mathcal{S}$ sampled species. However, we are interested not in any particular choices of species, but the behavior of the ecosystem when *typical* species are drawn from some random distribution. These typical ecosystems can be analyzed by calculating $\langle \log Z \rangle$, where the average is over our random draws of species. This average can be calculated through use of the replica trick,

$$\langle \log Z \rangle = \lim_{n \to 0} \frac{\langle Z^n \rangle - 1}{n}. \tag{S35}$$

In accordance with the replica trick, we consider n copies of our system, which share identical resident species but may differ in shot noise (encoded by the temperature β). Then, we treat n as a real number and consider the limit $n \to 0$, comparing our results to simulation to ensure this approximation holds. With n copies of our system, the average partition function is

$$\langle Z^n \rangle = \int \prod_{i,a} \left[dg_i^a e^{-\beta \sum_{i,a} F_i(g_i^a)} \right] \times$$
$$\prod_{\mu=1}^{S} \left\{ \prod_a \left[\int \frac{d\Delta_\mu^a d\hat{\Delta}_\mu^a}{2\pi} \theta(-\Delta_\mu^a) \right] e^{i \sum_a \hat{\Delta}_\mu^a \Delta_\mu^a} \left\langle e^{-i \tilde{X}_\mu \sum_a \hat{\Delta}_\mu^a} \right\rangle_{\tilde{X}_\mu} \times \right.$$
$$\left. \left\langle e^{i \sum_{i,a} \hat{\Delta}_\mu^a g_i^a \alpha_{\mu,i}} \right\rangle_{\alpha_{\mu,i}} \right\}, \tag{S36}$$

where a runs from 1 to n and indexes the replicas of our system. Next, let's average over our random draws of species. Suppose that the pure fitnesses $\tilde{X}_\mu$ are drawn from a normal distribution with mean $\chi/\mathcal{R}_0$ and variance $(\epsilon/\mathcal{R}_0)^2$. Recall that χ represents our fitness gauge, and will later be set to ensure that $\langle g_i \rangle = 0$. The factors of $1/\mathcal{R}_0$ account for the fact that the $\alpha_{\mu,i}$ in Eq. (S25) are $\sim 1/\mathcal{R}_0$ smaller than the corresponding terms in Refs. (1) and (10) due to the overall normalization of $\boldsymbol{\alpha}$. The $\tilde{X}_\mu$-dependent term then becomes

$$\left\langle e^{-i \tilde{X}_\mu \sum_a \hat{\Delta}_\mu^a} \right\rangle_{\tilde{X}_\mu} = \exp\left[-i \frac{\chi}{\mathcal{R}_0} \sum_a \hat{\Delta}_\mu^a - \frac{\epsilon^2}{2\mathcal{R}_0^2} \left(\sum_a \hat{\Delta}_\mu^a \right)^2 \right]. \tag{S37}$$

Now, let's consider the average over the strategy vectors. For binary resource strategies, the $\alpha_{\mu i}$ are defined as

$$\alpha_{\mu i} = \frac{\sigma_{\mu i}}{\sum_j \sigma_{\mu j}}, \tag{S38}$$

where $\sigma_{\mu i}$ are i.i.d. random variables equal to 1 with probability $\mathcal{R}_0/\mathcal{R}$ and 0 otherwise. Unlike the $\sigma_{\mu i}$, the $\alpha_{\mu i}$ are not independent for the same μ with different i's. However, we can approximate them as independent by separating out the dependent portion, provided that $\mathcal{R}_0 \gg 1$:

$$\alpha_{\mu i} = \frac{\sigma_{\mu i}}{\mathcal{R}_0 + b_\mu \sqrt{\mathcal{R}_0}} \tag{S39}$$

$$\approx \frac{\sigma_{\mu i}}{\mathcal{R}_0}\left(1 - \frac{b_\mu}{\sqrt{\mathcal{R}_0}}\right). \tag{S40}$$

The b_μ are mean-zero $\mathcal{O}(1)$ random variables representing the fluctuations in the total number of resources used by each species. While not technically independent of the $\sigma_{\mu i}$, this dependence would contribute to terms with smaller powers of $\mathcal{R}_0$ and can thus be ignored. So, we consider their averages separately:

$$\left\langle e^{i\sum_{i,a}\hat{\Delta}^a_\mu g^a_i \alpha_{\mu,i}}\right\rangle_{\alpha_{\mu,i}} \approx \left\langle \prod_i \left[1 - \frac{\mathcal{R}_0}{\mathcal{R}} + \frac{\mathcal{R}_0}{\mathcal{R}} e^{i\sum_a \hat{\Delta}^a_\mu g^a_i/\mathcal{R}_0} e^{-i\sum_a \hat{\Delta}^a_\mu g^a_i b_\mu/\mathcal{R}_0^{3/2}}\right]\right\rangle_{b_\mu}$$

$$\approx \left\langle \prod_i \exp\left[i\frac{1}{\mathcal{R}}\sum_a \hat{\Delta}^a_\mu g^a_i \left(1 - \frac{b_\mu}{\mathcal{R}_0}\right) - \frac{(1-\mathcal{R}_0/\mathcal{R})}{2\mathcal{R}_0\mathcal{R}}\left(\sum_a \hat{\Delta}^a_\mu g^a_i\right)^2\right]\right\rangle_{b_\mu} \tag{S41}$$

$$= \left\langle e^{-i\sum_{i,a}\hat{\Delta}^a_\mu g^a_i b_\mu/\mathcal{R}\sqrt{\mathcal{R}_0}}\right\rangle_{b_\mu} \prod_i \exp\left[i\frac{1}{\mathcal{R}}\sum_a \hat{\Delta}^a_\mu g^a_i - \frac{(1-\mathcal{R}_0/\mathcal{R})}{2\mathcal{R}\mathcal{R}_0}\left(\sum_a \hat{\Delta}^a_\mu g^a_i\right)^2\right] \tag{S42}$$

$$\approx \exp\left[-\frac{(\sum_{i,a}\hat{\Delta}^a_\mu g^a_i)^2}{2R^2\mathcal{R}_0}\right] \prod_i \exp\left[i\frac{1}{\mathcal{R}}\sum_a \hat{\Delta}^a_\mu g^a_i - \frac{(1-\mathcal{R}_0/\mathcal{R})}{2\mathcal{R}\mathcal{R}_0}\left(\sum_a \hat{\Delta}^a_\mu g^a_i\right)^2\right] \tag{S43}$$

$$\approx \prod_i \exp\left[i\frac{1}{\mathcal{R}}\sum_a \hat{\Delta}^a_\mu g^a_i - \frac{(1-\mathcal{R}_0/\mathcal{R})}{2\mathcal{R}\mathcal{R}_0}\left(\sum_a \hat{\Delta}^a_\mu g^a_i\right)^2\right]. \tag{S44}$$

Effectively, this approximation treats $\alpha_{\mu i} \approx \sigma_{\mu_i}/\mathcal{R}_0$. Finally, to bring our answer in line with Refs. (1) and (10), we rescale variables $\Delta^a_\mu \to \Delta^a_\mu/\mathcal{R}_0$ and $\hat{\Delta}^a_\mu \to \mathcal{R}_0\hat{\Delta}^a_\mu$. The final partition function is

$$\langle Z^n \rangle = \int \prod_{i,a}\left[dg^a_i e^{-\beta\sum_{i,a}F_i(g^a_i)}\right] \prod_{\mu=1}^{S}\left\{\prod_a \left[\int \frac{d\Delta^a_\mu d\hat{\Delta}^a_\mu}{2\pi}\theta(-\Delta^a_\mu)\right] \times \right.$$
$$\exp\left[i\sum_a \hat{\Delta}^a_\mu\left(\Delta^a_\mu + \frac{\mathcal{R}_0}{\mathcal{R}}\sum_i g^a_i - \chi\right) - \frac{\epsilon^2}{2}\left(\sum_a \hat{\Delta}^a_\mu\right)^2 - \right.$$
$$\left.\left.\frac{\mathcal{R}_0(1-\mathcal{R}_0/\mathcal{R})}{2\mathcal{R}}\sum_i\left(\sum_a \hat{\Delta}^a_\mu g^a_i\right)^2\right]\right\}. \tag{S45}$$

This is the same as the partition function calculated in Refs. (1) and (10), except for the presence of χ. We can ignore χ until later in the calculation (for example, by absorbing it into the definition of Δ_μ^a, which causes it to appear only within the Heaviside function), and re-introduce it only when we require that $\langle g_i \rangle = 0$.

2.3 Introducing First-Step Mutations

The above partition function is valid for an ecosystem where all species are sampled randomly. However, in our "simultaneous assembly" approximation of first-step mutation, we would like to include two sampled species which are very related to each other, differing only by a single knock-out mutation. We will index the parent and mutant species P and M; they have the same resource consumption strategy, except that the parent uses resource 1, while the mutant's capacity to use resource 1 is lowered by a factor of γ. For a full knock-out mutation which preserves our assumption about binary resource use, $\gamma = 1$, but this more general type of mutation will aid us when interpreting our conclusions later. In particular, we will find that the magnitude of the phenotypic change, $\|\Delta\boldsymbol{\alpha}\| \approx \gamma/\mathcal{R}_0$, is most important when predicting its impact on the community. We assume the mutant's pure fitness is increased by a total amount ΔX relative to its parent (which can be negative to indicate an overall cost to the mutation). With the parent-mutant pair accounted for, the partition function becomes

$$\begin{aligned}
\langle Z^n \rangle = &\int \prod_{i,a} \left[dg_i^a e^{-\beta \sum_{i,a} F_i(g_i^a)} \right] \prod_{\mu=1}^{S-1} \left\{ \prod_a \left[\int \frac{d\Delta_\mu^a d\hat{\Delta}_\mu^a}{2\pi} \theta(-\Delta_\mu^a) \right] \times \right. \\
&\exp\left[i \sum_a \hat{\Delta}_\mu^a \left(\Delta_\mu^a + \frac{\mathcal{R}_0}{\mathcal{R}} \sum_i g_i^a - \chi \right) - \frac{\epsilon^2}{2} \left(\sum_a \hat{\Delta}_\mu^a \right)^2 - \right. \\
&\left.\left. \frac{\mathcal{R}_0(1-\mathcal{R}_0/\mathcal{R})}{2\mathcal{R}} \sum_i \left(\sum_a \hat{\Delta}_\mu^a g_i^a \right)^2 \right] \right\} \times \\
&\prod_a \left[\int \frac{d\Delta_P^a d\hat{\Delta}_P^a}{2\pi} \frac{d\Delta_P^a d\hat{\Delta}_P^a}{2\pi} \theta(-\Delta_P^a) \theta(-\Delta_M^a) \right] \\
&\exp\left\{ i \sum_a \hat{\Delta}_P^a \left(\Delta_P^a + \frac{\mathcal{R}_0}{\mathcal{R}} \sum_i g_i^a + \left(1 - \frac{\mathcal{R}_0}{\mathcal{R}} \right) g_1^a - \chi \right) + \right. \\
&i \sum_a \hat{\Delta}_M^a \left(\Delta_M^a + \frac{\mathcal{R}_0}{\mathcal{R}} \sum_i g_i^a + \left(1 - \gamma - \frac{\mathcal{R}_0}{\mathcal{R}} \right) g_1^a - \chi - \mathcal{R}_0 \Delta X \right) - \\
&\frac{\epsilon^2}{2} \left[\sum_a \left(\hat{\Delta}_P^a + \hat{\Delta}_M^a \right) \right]^2 - \frac{\mathcal{R}_0(1-\mathcal{R}_0/\mathcal{R})}{2\mathcal{R}} \sum_i \left[\sum_a \left(\hat{\Delta}_P^a + \hat{\Delta}_M^a \right) g_i^a \right]^2 + \\
&\left. \frac{\mathcal{R}_0(1-\mathcal{R}_0/\mathcal{R})}{2\mathcal{R}} \left[\sum_a \left(\hat{\Delta}_P^a + \hat{\Delta}_M^a \right) g_1^a \right]^2 \right\}. \qquad \text{(S46)}
\end{aligned}$$

(Note that the ΔX term has a factor of $\mathcal{R}_0$ to account for the rescaling we did earlier.) The next few steps of the calculation are a series of approximations which allow us to evaluate

this complicated integral, paralleling those in Refs. (1) and (10).

2.4 Saddle Point Approximations

For $\mathcal{R} \gg 1$, the central limit theorem dictates that the mean and variance of the equilibrium g_i (which encode the availabilities of each resource) should approach a deterministic value. Introducing the mutant-parent pair is a relatively small change in the ecosystem, so we expect that these deterministic values should be the same as those calculated in previous work, if the mutant-parent pair were not present. This motivates us to define replica-specific "order parameters"

$$m^a = \sum_i g_i^a, \tag{S47}$$

$$q^{ab} = \sum_i g_i^a g_i^b. \tag{S48}$$

As we did with the resource surplus Δ_μ^a, we can introduce these order parameters as new integration variables, defined via delta functions in their Fourier representation. This results in an integral

$$\begin{aligned}
\langle Z^n \rangle = &\int \prod_{a \le b} \left(\frac{dq^{ab} d\hat{q}^{ab}}{2\pi} \right) \prod_a \left(\frac{dm^a d\hat{m}^a}{2\pi} \right) \exp \left[i \left(\sum_{a \le b} q^{ab} \hat{q}^{ab} + \sum_a m^a \hat{m}^a \right) \right] \times \\
&\prod_{i=2}^{\mathcal{R}} \left\{ \int_{-\infty}^1 \prod_a dg_i^a \exp \left[-\sum_a \beta F_i(g_i^a) - i \sum_a \hat{m}^a g_i^a - i \sum_{a \le b} \hat{q}^{ab} g_i^a g_i^b \right] \right\} \times \\
&\prod_{\mu=1}^{S-1} \left\{ \prod_a \left[\int \frac{d\Delta_\mu^a d\hat{\Delta}_\mu^a}{2\pi} \theta(-\Delta_\mu^a) \right] \times \right. \\
&\left. \exp \left[i \sum_a \hat{\Delta}_\mu^a \left(\Delta_\mu^a + \frac{\mathcal{R}_0}{\mathcal{R}} m^a - \chi \right) - \frac{1}{2} \sum_{a,b} \left(\frac{\mathcal{R}_0 (1 - \mathcal{R}_0/\mathcal{R})}{\mathcal{R}} q^{ab} + \epsilon^2 \right) \hat{\Delta}_\mu^a \hat{\Delta}_\mu^b \right] \right\} \\
&\times \prod_a \left[\int \frac{d\Delta_P^a d\hat{\Delta}_P^a}{2\pi} \frac{d\Delta_M^a d\hat{\Delta}_M^a}{2\pi} \theta(-\Delta_P^a) \theta(-\Delta_M^a) \int_{-\infty}^1 dg_1^a \right] \exp \left\{ \sum_a \beta F_1(g_1^a) \right. \\
&- i \sum_a \hat{m}^a g_1^a - i \sum_{a \le b} \hat{q}^{ab} g_1^a g_1^b + i \sum_a \hat{\Delta}_P^a \left(\Delta_P^a + \frac{\mathcal{R}_0}{\mathcal{R}} m^a + \left(1 - \frac{\mathcal{R}_0}{\mathcal{R}} \right) g_1^a - \chi \right) \\
&+ i \sum_a \hat{\Delta}_M^a \left(\Delta_M^a + \frac{\mathcal{R}_0}{\mathcal{R}} m^a + \left(1 - \gamma - \frac{\mathcal{R}_0}{\mathcal{R}} \right) g_R^a - \chi - \mathcal{R}_0 \Delta X \right) \\
&\left. - \frac{1}{2} \sum_{a,b} \left[\frac{\mathcal{R}_0 (1 - \mathcal{R}_0/\mathcal{R})}{\mathcal{R}} (q^{ab} - g_1^a g_1^b) + \epsilon^2 \right] \left(\hat{\Delta}_P^a \hat{\Delta}_P^b + 2 \hat{\Delta}_P^a \hat{\Delta}_M^b + \hat{\Delta}_M^a \hat{\Delta}_M^b \right) \right\}.
\end{aligned} \tag{S49}$$

So far, we have made no approximations from our initial partition function. Now, however, we invoke the saddle-point approximation, stating that the probability distribution for the

order parameters and their Fourier conjugates is so heavily peaked that their values are equivalent across replicas. In short, we assume

$$m^a = m, \tag{S50}$$

$$q^{aa} = q^D \tag{S51}$$

$$q^{ab} = q^O \text{ if } a \neq b, \tag{S52}$$

and likewise for the conjugate (hatted) order parameters. We recall that we must eventually choose our fitness gauge χ such that $m = 0$, a deviation from the analysis in Refs. (1) and (10) due to our choice of consumer resource model. Our saddle point approximation allows us to significantly simplify our integral, by eliminating the integrals over the replica-specific order parameters. This change decouples our large integral expression into a product of three *independent* integrals: one over the resource availabilities g_i for $i > 1$, one over the surpluses Δ_μ^a for all species besides the parent and the mutant, and the final integral over g_1 and the surpluses for the mutant and parent strains.

Because we assume a diverse, many-species ecosystem, the species besides the parent and mutant will be most important in setting the saddle-point values of our order parameters and their Fourier conjugates. Thus, we approximate that these saddle-point values are unchanged from the calculation in Refs. (1) and (10), which includes only the first two sets of integrals (i.e., those independent of the parent and mutant). While we will not restate their calculation here, we will summarize their results. If we assume that the values of q^D and q^O are similar –

$$q^D \approx q^O = q, \tag{S53}$$

$$q^D - q^O = \frac{\mathcal{R}}{\beta} x, \tag{S54}$$

then the saddle-point values of the conjugate variables are

$$\hat{m} \approx i\beta, \tag{S55}$$

$$\hat{q}^O \approx i \left(\frac{\beta}{x} \sqrt{\frac{q}{\mathcal{R}}} \right)^2, \tag{S56}$$

$$\hat{q}^D - \frac{\hat{q}^O}{2} \approx i\beta \frac{x-1}{2x}. \tag{S57}$$

These equations leave q and x undetermined, which can be solved for implicitly and approximated through numerical equations which we will discuss later.

We also make the approximation $q^O - g_1^a g_1^b \approx q^O$. The order parameter q is formed by adding together $g_i^a g_i^b$ for each of $\mathcal{R}$ resources, and we expect each of these quantities to be positive. So, the scale of $q - g_R^a g_R^b \sim q(\mathcal{R} - 1)/\mathcal{R} \sim q$. After making this approximation, let us also recall that $F_i(g_i^a) = \kappa_i \left[g_i^a + (g_i^a)^2/2 \right]$. Knowing that $m = 0$ and $\hat{m} \approx i\beta$ causes all the terms linearly dependent on the g_i^a to cancel out, besides the mutation-dependent g_1^a terms: thus, before considering the effects of the mutation, our system is invariant under rotation of the g_i^a's. Practically, this means that our initial choice of a knock-out mutation was arbitrary: a knock-in mutation, or any strategy change $\Delta\boldsymbol{\alpha}$ with the same overall

magnitude, can be redefined as a change in a single element through an appropriate rotation. Our results should therefore be valid for mutations which affect multiple resources simultaneously.

2.5 Joint Distribution of Δ_P and Δ_M

Now that we have decoupled our integrals through the saddle-point approximation, we are nearly ready to analyze the phenomenon of parent-mutant coexistence. To do this, we only care about the part of the partition function which depends on those strains. For simplicity, we will also at this point assume uniform resource supply ($\kappa_i = 1$); we will discuss at the end of our calculation what happens when this assumption is relaxed. With the saddle-point approximation for $\kappa_i = 1$, the portion of the integral in Eq. (S49) which depends on the parent and mutant strains is

$$\begin{aligned}
Z^n_{PM} = \prod_a \left[\int \frac{d\Delta^a_P d\hat{\Delta}^a_P}{2\pi} \frac{d\Delta^a_M d\hat{\Delta}^a_M}{2\pi} \theta(-\Delta^a_P)\theta(-\Delta^a_M) \int_{-\infty}^{1} dg^a_1 \right] \times \\
\exp\left\{ \sum_a \left[-\frac{\beta}{2x}(g^a_1)^2 \right] + \frac{1}{2}\left(\frac{\beta}{x}\sqrt{\frac{q}{\mathcal{R}}} \sum_a g^a_1 \right)^2 \right. \\
+ i \sum_a \hat{\Delta}^a_P \left(\Delta^a_P + \frac{\mathcal{R}_0}{\mathcal{R}} m + \left(1 - \frac{\mathcal{R}_0}{\mathcal{R}}\right) g^a_1 - \chi \right) \\
+ i \sum_a \hat{\Delta}^a_M \left(\Delta^a_M + \frac{\mathcal{R}_0}{\mathcal{R}} m + \left(1 - \gamma - \frac{\mathcal{R}_0}{\mathcal{R}}\right) g^a_1 - \chi - \mathcal{R}_0 \Delta X \right) \\
- \frac{\mathcal{R}_0(1-\mathcal{R}_0/\mathcal{R})}{2\beta} x \sum_a \left(\hat{\Delta}^a_P + \hat{\Delta}^a_M \right)^2 \\
\left. - \frac{1}{2} \sum_{a,b} \left[\frac{\mathcal{R}_0(1-\mathcal{R}_0/\mathcal{R})}{\mathcal{R}} q + \epsilon^2 \right] \left(\hat{\Delta}^a_P \hat{\Delta}^b_P + 2\hat{\Delta}^a_P \hat{\Delta}^b_M + \hat{\Delta}^a_M \hat{\Delta}^b_M \right) \right\}. \qquad \text{(S58)}
\end{aligned}$$

Next, we perform a change of variables to $\hat{\Delta}^a_\pm = \hat{\Delta}^a_P \pm \hat{\Delta}^a_M$:

$$\begin{aligned}
Z^n_{PM} = \prod_a \left[\int \frac{d\Delta^a_P d\Delta^a_M}{4\pi} \frac{d\hat{\Delta}^a_+ d\hat{\Delta}^a_-}{4\pi} \theta(-\Delta^a_P)\theta(-\Delta^a_M) \int_{-\infty}^{1} dg^a_1 \right] \times \\
\exp\left\{ \sum_a \left[-\frac{\beta}{2x}(g^a_1)^2 \right] + \frac{1}{2}\left(\frac{\beta}{x}\sqrt{\frac{q}{\mathcal{R}}} \sum_a g^a_1 \right)^2 \right. \\
+ i \sum_a \hat{\Delta}^a_+ \left(\frac{\Delta^a_P + \Delta^a_M}{2} + \frac{\mathcal{R}_0}{\mathcal{R}} m + \left(1 - \frac{\gamma}{2} - \frac{\mathcal{R}_0}{\mathcal{R}}\right) g^a_1 - \chi - \mathcal{R}_0 \frac{\Delta X}{2} \right) \\
+ i \sum_a \hat{\Delta}^a_- \left(\frac{\Delta^a_P - \Delta^a_M}{2} + \frac{\gamma}{2} g^a_1 + \mathcal{R}_0 \frac{\Delta X}{2} \right) - \frac{\mathcal{R}_0(1-\mathcal{R}_0/\mathcal{R})}{2\beta} x \sum_a \left(\hat{\Delta}^a_+ \right)^2 \\
\left. - \frac{1}{2} \sum_{a,b} \left[\frac{\mathcal{R}_0(1-\mathcal{R}_0/\mathcal{R})}{\mathcal{R}} q + \epsilon^2 \right] \left(\sum_a \hat{\Delta}^a_+ \right)^2 \right\}. \qquad \text{(S59)}
\end{aligned}$$

We now aim to decouple the replicas, by eliminating terms which involve more than one replica. All these terms are of the form $(\sum_a f^a)^2$, where f^a is some quantity which depends only on one replica. We can decouple these replicas using the identity

$$\exp\left(\frac{1}{2}(Cx)^2\right) = \int d\omega \frac{e^{-\omega^2/2}}{\sqrt{2\pi}} e^{Cx\omega}. \tag{S60}$$

Thus, we can decouple the replicas at the cost of introducing an additional Gaussian integration variable per square term. We can now drop the a indices and replace them with a simple power to n:

$$\begin{aligned} Z_{PM}^n = \int d\omega_1 d\omega_2 \Bigg\{ \int \frac{d\Delta_P d\Delta_M}{4\pi} \frac{d\hat{\Delta}_+ d\hat{\Delta}_-}{4\pi} \theta(-\Delta_P)\theta(-\Delta_M) \int_{-\infty}^{1} dg_1 \\ \times \exp\Bigg[-\frac{\beta}{2x} g_1^2 + \omega_1 \frac{\beta}{x}\sqrt{\frac{q}{\mathcal{R}}} g_1 - \frac{\mathcal{R}_0(1-\mathcal{R}_0/\mathcal{R})}{2\beta} x\hat{\Delta}_+^2 \\ + i\hat{\Delta}_+ \left(\frac{\Delta_P + \Delta_M}{2} + \frac{\mathcal{R}_0}{\mathcal{R}} m + \left(1 - \frac{\gamma}{2} - \frac{\mathcal{R}_0}{\mathcal{R}}\right) g_1 + \omega_2 V_{\text{tot}}^{1/2} - \chi - \mathcal{R}_0 \frac{\Delta X}{2} \right) \\ + i\hat{\Delta}_- \left(\frac{\Delta_P - \Delta_M}{2} + \frac{\gamma}{2} g_1 + \mathcal{R}_0 \frac{\Delta X}{2} \right) \Bigg] \Bigg\}^n \frac{e^{-(\omega_1^2+\omega_2^2)/2}}{2\pi}. \end{aligned} \tag{S61}$$

Here, we have defined $V_{\text{tot}} = q\mathcal{R}_0(1 - \mathcal{R}_0/\mathcal{R})/\mathcal{R} + \epsilon^2$. This parameter, defined as ψ^2 in Refs. (1) and (10), represents the total variance in fitness among sampled species from both strategy and pure fitness. As we will demonstrate later, the integration variables ω_1 and ω_2 do in fact have physical meaning: ω_1 is related to the invasion fitness of the mutant, while ω_2 is related to the abundance of the parent. Next, we perform the integral over g_1. To do so, we assume that most of the weight of the integral is concentrated near small g, meaning that we can approximate the integration bounds as $-\infty$ to ∞ rather than $-\infty$ to 1. We also condense all of the resulting terms which do not depend on $\hat{\Delta}_+$ or $\hat{\Delta}_-$ into a constant term c.

We can write the result as

$$\begin{aligned} Z_{PM}^n = \int d\omega_1 d\omega_2 \Bigg\{ \int \frac{d\Delta_P d\Delta_M}{4\pi} \frac{d\hat{\Delta}_+ d\hat{\Delta}_-}{4\pi} \theta(-\Delta_P)\theta(-\Delta_M) \\ \times \exp\left[-\frac{1}{2} \mathbf{\Delta} \mathbf{M} \mathbf{\Delta}^t + i\mathbf{\Delta}\cdot\mathbf{v} + c \right] \Bigg\}^n \frac{e^{-(\omega_1^2+\omega_2^2)/2}}{2\pi}, \end{aligned} \tag{S62}$$

where

$$\mathbf{\Delta} = \begin{bmatrix} \hat{\Delta}_+ & \hat{\Delta}_- \end{bmatrix}, \tag{S63}$$

$$\mathbf{M} = \frac{x}{\beta} \begin{bmatrix} A & B \\ B & C \end{bmatrix}, \tag{S64}$$

$$\mathbf{v} = \begin{bmatrix} \frac{\Delta_P + \Delta_M}{2} + D & \frac{\Delta_P - \Delta_M}{2} + E \end{bmatrix}, \tag{S65}$$

and we define

$$A = \mathcal{R}_0 \left(1 - \frac{\mathcal{R}_0}{\mathcal{R}}\right) + \left(1 - \frac{\gamma}{2} - \frac{\mathcal{R}_0}{\mathcal{R}}\right)^2, \tag{S66}$$

$$B = \frac{\gamma}{2}\left(1 - \frac{\gamma}{2} - \frac{\mathcal{R}_0}{\mathcal{R}}\right), \tag{S67}$$

$$C = \frac{\gamma^2}{4}, \tag{S68}$$

$$D = \omega_2 V_{\text{tot}}^{1/2} + \frac{\mathcal{R}_0}{\mathcal{R}} m + \omega_1 \sqrt{\frac{q}{\mathcal{R}}}\left(1 - \frac{\gamma}{2} - \frac{\mathcal{R}_0}{\mathcal{R}}\right) - \chi - \mathcal{R}_0 \frac{\Delta X}{2}, \tag{S69}$$

$$E = \mathcal{R}_0 \frac{\Delta X}{2} + \omega_1 \frac{\gamma}{2}\sqrt{\frac{q}{\mathcal{R}}}. \tag{S70}$$

We can now perform the integrals over $\hat{\Delta}_+$ and $\hat{\Delta}_-$. This yields

$$Z_{PM}^n = \int d\omega_1 d\omega_2 \left[\frac{1}{\sqrt{\det \mathbf{M}}} \int \frac{d\Delta_P d\Delta_M}{4\pi} \theta(-\Delta_P)\theta(-\Delta_M) \times \exp\left(-\frac{1}{2}\mathbf{v}\mathbf{M}^{-1}\mathbf{v}^t + c\right)\right]^n \frac{e^{-(\omega_1^2+\omega_2^2)/2}}{2\pi}. \tag{S71}$$

Now, let's utilize the replica trick limit $n \to 0$. Again following the methodology in Refs. (1) and (10), this can be used to recover the joint probability distribution $\rho(\Delta_P, \Delta_M)$:

$$\lim_{n\to 0} Z_{PM}^n = \int_{-\infty}^{0} d\Delta_P d\Delta_M \int d\omega_1 d\omega_2 \frac{e^{-(\omega_1^2+\omega_2^2)/2}}{2\pi} \times \frac{\exp\left(-\frac{1}{2}\mathbf{v}\mathbf{M}^{-1}\mathbf{v}^t\right)}{\int_{-\infty}^{0} d\Delta_P' d\Delta_M' \exp\left(-\frac{1}{2}\mathbf{v}'\mathbf{M}^{-1}\mathbf{v}'^t\right)}, \tag{S72}$$

$$= \int_{-\infty}^{0} d\Delta_P d\Delta_M \rho(\Delta_P, \Delta_M). \tag{S73}$$

Recall that $\mathbf{M}^{-1} \sim \beta$, an inverse temperature which scales with the number of cells in our community, reflecting the shot noise in our population at any given time. If we assume that our population is always exactly at equilibrium (i.e., is large enough that shot noise is negligible), we can take the $\beta \to \infty$ limit, in which case the exponentials involving $\mathbf{M}^{-1}$ will be very strongly peaked. In this limit, we can therefore approximate the ratio in Eq. (S72) as a delta function:

$$\rho(\Delta_P, \Delta_M) = \int d\omega_1 d\omega_2 \frac{e^{-(\omega_1^2+\omega_2^2)/2}}{2\pi} \delta(\Delta_P - \Delta_P^*)\delta(\Delta_M - \Delta_M^*), \tag{S74}$$

where $\Delta_P^*(\omega_1, \omega_2)$ and $\Delta_M^*(\omega_1, \omega_2)$ are the non-positive values of Δ_P and Δ_M that minimize $\mathbf{v}\mathbf{M}^{-1}\mathbf{v}^t$ for a given ω_1 and ω_2.

2.6 Parent-Mutant Coexistence Probability

The parent-mutant coexistence probability is encoded in the joint distribution $\rho(\Delta_P, \Delta_M)$:

$$\mathbb{P}[\text{Both survive}] = \int d\omega_1 d\omega_2 \frac{e^{-(\omega_1^2+\omega_2^2)/2}}{2\pi} \delta(\Delta_P^*(\omega_1, \omega_2))\delta(\Delta_M^*(\omega_1, \omega_2)). \tag{S75}$$

So, the next step is to uncover the functional forms of Δ_P^* and Δ_M^*, which represent the values of Δ_P and Δ_M that minimize $\mathbf{v}\mathbf{M}^{-1}\mathbf{v}^t$, constrained such that both Δ_P and Δ_M are non-positive. The matrix $\mathbf{M}$ and its inverse are positive definite, so this is a convex optimization problem, and either the unconstrained minimum lies in the interior of the allowed region, or the function is optimized on the boundary. If the global minimum happens to be within the allowed region, corresponding to both the parent and the mutant being extinct, these quantities are simple:

$$\Delta_P^*\big|_{\Delta_P^*<0,\ \Delta_M^*<0} = -D - E, \tag{S76}$$

$$\Delta_M^*\big|_{\Delta_P^*<0,\ \Delta_M^*<0} = -D + E. \tag{S77}$$

This unconstrained minimum will be in the allowed region if both of these quantities are negative, which occurs when $D > |E|$. We see that when both the parent and mutant are extinct, D tells us the resource deficit averaged between the parent and mutant, while $2E$ is the difference in resource deficit between mutant and parent. If the average resource deficit is too large, then neither strain can survive, thus

$$\mathbb{P}[\text{Both die}] = \mathbb{P}[D > |E|]. \tag{S78}$$

If the unconstrained minimum lies outside the allowed region, then the function will be minimized on the boundary of the allowed region, when one or both of the parent and mutant are alive. For example, if the mutant survives and the parent goes extinct, the minimum will be found at $\Delta_M^* = 0$ and

$$\Delta_P^*\big|_{\Delta_P^*<0,\ \Delta_M^*=0} = \frac{2B(D+E) - 2(CD+AE)}{A - 2B + C}, \tag{S79}$$

$$\approx -2E + \frac{2}{A}(B - C)(D - E). \tag{S80}$$

This expansion comes from the fact that $A \sim \mathcal{R}_0$ (provided that $\mathcal{R}_0$ does not get too close to $\mathcal{R}$), while $B,\ C \sim 1$. Similarly, if the mutant goes extinct while the parent survives, we find

$$\Delta_M^*\big|_{\Delta_P^*=0,\ \Delta_M^*<0} = \frac{2E(A+B) - 2D(B+C)}{A + 2B + C}, \tag{S81}$$

$$\approx 2E - \frac{2}{A}(B + C)(D + E). \tag{S82}$$

The last possibility is that the optimum lies on the corner of the allowed region, where the parent and mutant coexist (so $\Delta_P^* = \Delta_M^* = 0$). We can find this probability by requiring

that each of these three potential optima are outside of the allowed region, which occurs when

$$\mathbb{P}[\text{Both survive}] \approx \mathbb{P}[BE + CD < -|AE - BD - CE|,\ D < |E|]. \tag{S83}$$

Recalling that $A \sim \mathcal{R}_0 \gg 1$, the AE term dominates unless $|E| \ll 1$, meaning that E must be small for the inequality to be possible. (Note that the $\mathcal{R}_0 \Delta X$ term is not actually $\mathcal{O}(\mathcal{R}_0)$, because the natural fitness scale for a mutant turns out to be $\Delta X \sim \mathcal{R}_0^{-3/2}$.) When this is the case, the BE and CE terms will be even smaller, allowing us to safely ignore them:

$$\mathbb{P}[\text{Both survive}] \approx \mathbb{P}[CD < -|AE - BD|,\ D < |E|], \tag{S84}$$

$$= \mathbb{P}[CD < -|AE - BD|]. \tag{S85}$$

We have eliminated the second condition because it is now redundant with the first: since $C > 0$, the first inequality requires $D < 0$, trivially satisfying $D < |E|$. Finally, let's recall that in our simultaneous assembly approximation of mutant invasion (Appendix 1.3), we must condition on the probability that the mutant survives:

$$\mathbb{P}_{\text{coex}} \approx \frac{\mathbb{P}[\text{Both survive}]}{\mathbb{P}[\text{Mutant survives}]}, \tag{S86}$$

$$\approx \frac{\mathbb{P}[CD < -|AE - BD|]}{\mathbb{P}[E > D, E > 0]}. \tag{S87}$$

In principle, this is a quantity that can be calculated numerically. However, this result is difficult to intuitively interpret in this form, and it still depends on the saddle-point order parameter q. Nonetheless, we can immediately make two meaningful conclusions. If we send the number of resources consumed by a typical organism $\mathcal{R}_0 \to \infty$, then A becomes large and $\mathbb{P}_{\text{coex}} \to 0$. Likewise, if we send the effect size of our mutation $\gamma \to 0$, then $\frac{|CD|}{|AE-BD|} \sim \gamma \to 0$, and thus $\mathbb{P}_{\text{coex}} \to 0$. Since we are considering a mutation whose effect size is comparable to changing one resource's uptake rate by a factor of γ, both of these observations suggest the same thing: if the phenotypic effect of a mutation is too small, mutant-parent coexistence is impossible. This phenomenon is a signature of "mesoscopic" mutation, suggesting that our results may not be captured by models of mutations with infinitesimal phenotypic effects, such as adaptive dynamics approaches (11, 12).

Supplementary Note 3: Scaling Analysis of First-Step Mutations

In this section, we aim to restore physical intuition to our replica-theoretic results, finding approximate expressions which indicate how the frequency of inter-niche evolution scales with community parameters such as the number of surviving species.

3.1 Sampling Depth and Fitness Gauge

We must utilize the saddle-point results for an assembled community without mutation, largely from Refs. (1) and (10), to calculate our order parameters m and q. However, we opt for a different set of independent variables, defining our assembly process in terms

of the number of sampled species $\mathcal{S}$ and the typical number of surviving species $\mathcal{S}^*$. The community assembly parameter ϵ, which describes the variation in pure fitness among sampled species, will then be defined implicitly as a function of these parameters. This change of variables is helpful because $\mathcal{S}^*$ is a measurable property of an experimental ecosystem, while ϵ describes a property of *sampled* species, which may be very different from typical surviving species and is thus more difficult to access experimentally.

With this set of variables, a natural ratio to consider is the "sampling permissivity" $\mathcal{S}^*/\mathcal{S}$, roughly the probability that a new independently sampled species would be able to invade the ecosystem. It turns out that it will be useful to define an effective "sampling depth" λ, which is a function of $\mathcal{S}^*/\mathcal{S}$:

$$\lambda \equiv \operatorname{erf}^{-1}\left(1 - 2\frac{\mathcal{S}^*}{\mathcal{S}}\right)\sqrt{2}. \tag{S88}$$

For an undersampled system ($\mathcal{S}^*/\mathcal{S} \to 1$), the sampling depth $\lambda \to -\infty$; for an oversampled system ($\mathcal{S}^*/\mathcal{S} \to 0$), we have $\lambda \to \infty$. From the saddle-point results of Refs. (1) and (10) (which we do not re-derive here), we know that

$$m = \frac{\mathcal{R}}{\mathcal{R}_0}\left(V_{\text{tot}}^{1/2}\lambda + \chi\right). \tag{S89}$$

Recall that χ sets our choice of fitness gauge: the uptake budget of sampled species is drawn with mean $\chi/\mathcal{R}_0$ and standard deviation $\epsilon/\mathcal{R}_0$. But in our community assembly approximations, we have chosen our gauge such that $\langle g_i \rangle = m/\mathcal{R} = 0$. This implies that

$$\chi = -V_{\text{tot}}^{1/2}\lambda. \tag{S90}$$

As we sample more species and λ increases, the uptake budgets or pure fitnesses of surviving species will be further and further into the high-fitness tail of the distribution. Since the typical pure fitness of surviving species is nearly zero in our fitness gauge, χ must become more negative as λ increases to compensate for the increased sampling. In the consumer resource model analyzed in Refs. (1) and (10) (where $\chi = 0$), an increase in sampling depth results in an overall increase in m: as oversampled species become higher and higher in pure fitness, the typical resource becomes less valuable to consume. This effect causes resource knock-out mutations to be more beneficial on average than knock-in mutations in the oversampled limit. In our model, we adjust the "zero" of pure fitness to compensate for this effect, such that $m = 0$ regardless of λ.

For future sections, it is helpful to define a function $I(\lambda)$ such that

$$I(\lambda) = \frac{1+\lambda^2}{2}\operatorname{erfc}\left(\frac{\lambda}{\sqrt{2}}\right) - \frac{\lambda}{\sqrt{2\pi}}e^{-\lambda^2/2}. \tag{S91}$$

3.2 Niche Saturation and σ_{inv}

From saddle-point results in Refs. (1) and (10), we can write expressions for V_{tot}, q, and ϵ in terms of quantities we have already defined, as well as variance in resource supply

$\sigma_K^2 \equiv \mathrm{Var}(\kappa) = \mathrm{Var}(K)/\overline{K}^2$:

$$V_{\mathrm{tot}}^{1/2} = \left(1 - \frac{\mathcal{R}_0}{\mathcal{R}}\right)\left(\frac{2\left(1 - \frac{S}{\mathcal{R}}I(\lambda)\right)}{-\frac{S}{\mathcal{R}}I'(\lambda)} - \lambda\right), \tag{S92}$$

$$q = V_{\mathrm{tot}}\frac{S}{\mathcal{R}}I(\lambda)\frac{\mathcal{R}}{\mathcal{R}_0(1-\mathcal{R}_0/\mathcal{R})} + \mathcal{R}\sigma_K^2\left(1 - \frac{\mathcal{S}^*}{\mathcal{R}}\right)^2, \tag{S93}$$

$$\epsilon^2 = V_{\mathrm{tot}}\left(1 - \frac{S}{\mathcal{R}}I(\lambda)\right) - \mathcal{R}_0\sigma_K^2\left(1 - \frac{\mathcal{R}_0}{\mathcal{R}}\right)\left(1 - \frac{\mathcal{S}^*}{\mathcal{R}}\right)^2. \tag{S94}$$

The order parameter q describes the total variance in resource availability, allowing us to find the distribution of the resource availabilities h_i: a Gaussian with mean 1 and variance $q/\mathcal{R}$. A mutation changing use of a single resource by a factor γ will have an invasion fitness with magnitude $\gamma|1-h_i|/\mathcal{R}_0$. This leads us to define the quantity

$$\sigma_{\mathrm{inv}} \equiv \frac{\gamma}{\mathcal{R}_0}\sqrt{\frac{q}{\mathcal{R}}}, \tag{S95}$$

which describes the typical magnitude of the invasion fitness of a strategy mutant $\boldsymbol{\alpha} \to \boldsymbol{\alpha} + \Delta\boldsymbol{\alpha}$, where $||\Delta\boldsymbol{\alpha}|| \approx \gamma/\mathcal{R}_0$. (For example, for a complete knock-out mutant for a system with binary resource use, we have $\gamma = 1$.)

While these equations completely determine all the variables we have defined, they are difficult to interpret physically, having complicated dependence on the ratios $\mathcal{S}/\mathcal{R}$ and $\mathcal{R}_0/\mathcal{R}$ as well as the sampling depth λ. We can make progress by considering how these equations depend on the *niche saturation* $\mathcal{S}^*/\mathcal{R} \leq 1$, describing how close to maximum capacity we expect our final ecosystem to be. By rewriting $\mathcal{S}/\mathcal{R} = (\mathcal{S}/\mathcal{S}^*)(\mathcal{S}^*/\mathcal{R})$, we separate out the parts of these equations that depend on the sampling depth λ (or equivalently, $\mathcal{S}^*/\mathcal{S}$) from the niche saturation $\mathcal{S}^*/\mathcal{R}$. (Technically, rather than using the value of $\mathcal{S}^*$ for any particular ecosystem as an input parameter, we use its expectation $\langle \mathcal{S}^* \rangle$ over random ecosystems with the same underlying parameters. Since these values tend to be close in practice, as shown in Figure 2B, we will simply write $\mathcal{S}^*$ in our scaling relationships.)

To simplify our results, it is helpful to expand our expressions in the undersampled and oversampled limits ($\lambda \to \pm\infty$). It is worth noting that the $\lambda \to -\infty$ limit for fixed $\mathcal{S}^*/\mathcal{R}$ does not correspond to a physical community assembly process: the expression for ϵ in Eqn. (S94) must be positive, but $(\mathcal{S}/\mathcal{R}) \cdot I(\lambda) \to \infty$ as $\lambda \to -\infty$, making the RHS of that equation negative. Effectively, this means that in order to sample species such that a desired fraction of niches are filled, a minimum fraction of sampled species must go extinct in the final ecosystem (on average). However, many quantities we are interested in are still well-defined in the $\lambda \to -\infty$ limit, and indeed scale similarly to the $\lambda \to \infty$ limit: we use this to demonstrate an overall lack of dependence on sampling depth.

We first quote results for $\sigma_K^2 = 0$. From standard expansions of the error function, we obtain

$$\sigma_{\mathrm{inv}} \approx \begin{cases} \gamma\left(1 - \frac{\mathcal{S}^*}{\mathcal{R}}\right)\sqrt{\frac{\mathcal{R}-\mathcal{R}_0}{\mathcal{S}^*\mathcal{R}_0^3}} & \text{when } \lambda \to -\infty, \\ \gamma\sqrt{2}\left(1 - \frac{\mathcal{S}^*}{\mathcal{R}}\right)\sqrt{\frac{\mathcal{R}-\mathcal{R}_0}{\mathcal{S}^*\mathcal{R}_0^3}} & \text{when } \lambda \to \infty. \end{cases} \tag{S96}$$

Remarkably, the typical invasion fitness scale of a strategy mutation has very weak dependence on the sampling depth λ, changing only by a factor of $\sqrt{2}$ from the extremely undersampled to the extremely oversampled regimes. This result allows us to effectively drop out λ (and thus $\mathcal{S}^*/\mathcal{S}$) as a variable, which was very difficult to see from our saddle-point results above.

3.3 Scaling Analysis of $\mathbb{P}_{\text{coex}}$

We got our expression for σ_{inv} "for free" from the saddle-point value of q, without need to explicitly consider the impact of the parent and mutant on the ecosystem. The same is not true for the mutant-parent coexistence probability: to obtain an interpretable expression for $\mathbb{P}_{\text{coex}}$, we must start from our results in Appendix 2.5, and then undergo a similar procedure of plugging in saddle-point values in the $\lambda \to \pm\infty$ limits. Recall that in our two-ecosystem approximation, we have

$$\mathbb{P}_{\text{coex}} \approx \frac{\mathbb{P}[\text{Both survive}]}{\mathbb{P}[\text{Mutant survives}]}. \tag{S97}$$

To get some intuition, let's start with the simpler calculation of the denominator of this expression: the probability that the mutant species survives in our assembled ecosystem. From our earlier results, this is

$$\mathbb{P}_{\text{M lives}} = \mathbb{P}\Bigg[\Delta X + \mathcal{Z}_1\sigma_{\text{inv}} > 0, \\ -\mathcal{Z}_2 V_{\text{tot}}^{1/2} + V_{\text{tot}}^{1/2}\lambda + \mathcal{Z}_1 \frac{\sigma_{\text{inv}}\mathcal{R}_0}{\gamma}\left(1 - \gamma - \frac{\mathcal{R}_0}{\mathcal{R}}\right) - \mathcal{R}_0\Delta X < 0\Bigg], \tag{S98}$$

where $\mathcal{Z}_i$ are independent standard normal random variables. Note that we have added a negative sign to $\mathcal{Z}_2$; while this leaves its distribution unchanged, it will aid in interpretation. The first two terms in the second inequality scale with the fitness variation in all sampled species $V_{\text{tot}}^{1/2}$, while the remaining terms scale with the fitness change induced by the mutation. If the mutation results in a small change in the organism's overall fitness, the first two terms should dominate, so we can approximate

$$\mathbb{P}_{\text{M lives}} \approx \mathbb{P}\left[\Delta X + \mathcal{Z}_1\sigma_{\text{inv}} > 0,\ \mathcal{Z}_2 - \lambda > 0\right], \tag{S99}$$

$$\approx \frac{\mathcal{S}^*}{2S}\text{erfc}\left(-\frac{\Delta X}{\sigma_{\text{inv}}\sqrt{2}}\right). \tag{S100}$$

Each of these inequalities has a meaning which allows us to interpret $\mathcal{Z}_{1,2}$ as physical quantities. The first inequality represents the probability the mutant is beneficial relative to the parent: the chances that the deterministic fitness change ΔX combined with the stochastic fitness change $\mathcal{Z}_1\sigma_{\text{inv}}$ due to the strategy mutation is positive. The second inequality represents the requirement that the parent background the mutant arises from is well-adapted enough to survive in the population in the first place: only draws of $\mathcal{Z}_2$ which are greater than λ will survive in the population. Indeed, the probability that $\mathcal{Z}_2 > \lambda$ is simply $\mathcal{S}^*/\mathcal{S}$,

the probability a randomly chosen species would survive in the community. Neglecting the other terms in the second inequality neglects the possibility that the parent cannot survive in the initial population, but the mutant can; as discussed in Supplementary Note 2.1, we expect this outcome to be rare.

We posit that $\mathcal{Z}_2$ determines not only the survival of the parent species, but also its approximate relative abundance f_P. Past research (3) suggests that consumer resource models of this form yield a truncated Gaussian distribution of abundances, which (up to an overall normalization factor) coincides with the distribution of $\mathcal{Z}_2$ conditioned on being greater than λ. We can find this normalization factor by enforcing $\langle f_P | f_P > 0 \rangle = 1/\mathcal{S}^*$, which yields

$$f_P \approx \frac{2}{-\mathcal{S} I'(\lambda)} (\mathcal{Z}_2 - \lambda)\theta(\mathcal{Z}_2 - \lambda). \tag{S101}$$

In the oversampled limit $\lambda \gg 1$, the prefactor is approximately $\lambda/\mathcal{S}^*$. Our results in the next section confirm that using this relationship between $\mathcal{Z}_2$ and f_P yields reasonably accurate predictions.

Now, let's calculate the numerator of $\mathbb{P}_{\text{coex}}$, the probability that the mutant and parent will both be alive in our simultaneously assembled ecosystem. Again approximating that terms which scale with overall fitness variation $V_{\text{tot}}^{1/2}$ dominate terms which scale with mutant fitness change ΔX and σ_{inv}, we find that

$$\mathbb{P}_{\text{P,M live}} \approx \mathbb{P}\left[\frac{\gamma V_{\text{tot}}^{1/2}}{\mathcal{R}_0^2} (\mathcal{Z}_2 - \lambda) > \Delta X + \mathcal{Z}_1 \sigma_{\text{inv}} > \frac{\gamma(1 - \gamma - \frac{\mathcal{R}_0}{\mathcal{R}}) V_{\text{tot}}^{1/2}}{\mathcal{R}_0^2 (1 - \frac{\mathcal{R}_0}{\mathcal{R}})} (\mathcal{Z}_2 - \lambda) \right]. \tag{S102}$$

Let us analyze this probability using our physical interpretations of $\mathcal{Z}_1$ and $\mathcal{Z}_2$. The outer inequality only holds when $\mathcal{Z}_2 > \lambda$, again requiring that the parent species survives. The middle term is the leading contribution to the invasion fitness of the mutant, which is $\mathcal{O}(\mathcal{R}_0^{-3/2})$. However, the inequality bounds it between two quantities of size $\mathcal{O}(\mathcal{R}_0^{-2})$. So, we conclude that parent-mutant coexistence is achieved when the invasion fitness of the mutant is sufficiently small.

Since the allowed region for $\mathcal{Z}_1$ is smaller than its typical width by a factor of $1/\sqrt{\mathcal{R}_0}$, we can approximate its probability density as constant over the allowed region. This gives us

$$\mathbb{P}_{\text{P,M live}} \approx \frac{e^{-(\Delta X/\sigma_{\text{inv}})^2/2}}{\sqrt{2\pi}} \frac{\gamma^2 V_{\text{tot}}^{1/2}}{\sigma_{\text{inv}} \mathcal{R}_0^2 (1 - \frac{\mathcal{R}_0}{\mathcal{R}})} \int_\lambda^\infty (\mathcal{Z}_2 - \lambda) \frac{e^{-\mathcal{Z}_2^2/2}}{\sqrt{2\pi}} d\mathcal{Z}_2 \tag{S103}$$

$$= \frac{e^{-(\Delta X/\sigma_{\text{inv}})^2/2}}{\sqrt{2\pi}} \frac{\gamma^2 V_{\text{tot}}^{1/2}}{\sigma_{\text{inv}} \mathcal{R}_0^2 (1 - \frac{\mathcal{R}_0}{\mathcal{R}})} \frac{-I'(\lambda)}{2}. \tag{S104}$$

Finally, we divide by the probability the mutant lives, and consider the undersampled and oversampled limits when $\Delta X = 0$:

$$\mathbb{P}_{\text{coex}} \approx \begin{cases} \gamma \sqrt{\frac{2\mathcal{R}}{\pi \mathcal{S}^*}} \sqrt{\frac{1}{\mathcal{R}_0 (1 - \mathcal{R}_0/\mathcal{R})}} & \text{when } \lambda \to -\infty, \\ \gamma \sqrt{\frac{\mathcal{R}}{\pi \mathcal{S}^*}} \sqrt{\frac{1}{\mathcal{R}_0 (1 - \mathcal{R}_0/\mathcal{R})}} & \text{when } \lambda \to \infty. \end{cases} \tag{S105}$$

Once again, the dependence on the sampling depth is nearly negligible, contributing only an $\mathcal{O}(1)$ numerical factor. Note, however, that this result is different from that in the main text in Equation (9). This is because in our theoretical calculation, we arbitrarily chose the identity of the parent species and the mutation, effectively giving each surviving species and each mutation an equal chance of being selected. In reality, each surviving species produces mutations at a rate proportional to its abundance, and mutations have a probability proportional to their invasion fitness of surviving genetic drift (13). In the next section, we will discuss how to correct for this difference.

3.4 Dependence of $\mathbb{P}_{\mathrm{coex}}$ on Invasion Fitness and Abundance

Let us reframe our expression for $\mathbb{P}_{\mathrm{coex}}$ in terms of the abundance f_P of the parent and the invasion fitness s_{inv} of the mutant, relating them to $\mathcal{Z}_1$ and $\mathcal{Z}_2$ as we did earlier. We can read off the invasion fitness of a beneficial mutant as the saddle-point value of Δ_M^* when Δ_P is fixed at zero, representing the initial growth rate of the mutant *before* it has a chance to invade:

$$s_{\mathrm{inv}} \approx \Delta X + \mathcal{Z}_1 \sigma_{\mathrm{inv}} + \frac{\gamma}{\mathcal{R}_0^2} V_{\mathrm{tot}}^{1/2} (\mathcal{Z}_2 - \lambda). \tag{S106}$$

We can plug this expression into our earlier probability that both the mutant and parent live to find that

$$\mathbb{P}_{\mathrm{P,M\ live}} \approx \mathbb{P}\left[0 < s_{\mathrm{inv}} < \frac{\gamma^2 V_{\mathrm{tot}}^{1/2}}{\mathcal{R}_0^2 (1 - \mathcal{R}_0/\mathcal{R})} (\mathcal{Z}_2 - \lambda)\right]. \tag{S107}$$

Recall that $\mathcal{Z}_2 - \lambda$ is proportional to the abundance of the parent, as we calculated in Eq. (S101) . While $\mathcal{Z}_2$ is not independent of s_{inv}, its contribution to s_{inv} is small, so approximating them as independent is reasonable. Plugging in this relationship, we find that

$$\mathbb{P}_{\mathrm{coex}} \approx \mathbb{P}\left[s_{\mathrm{inv}} < -I'(\lambda) \frac{\gamma^2 V_{\mathrm{tot}}^{1/2} \mathcal{S}}{2\mathcal{R}_0^2 (1 - \mathcal{R}_0/\mathcal{R})} f_P\right], \tag{S108}$$

$$= \mathbb{P}\left[s_{\mathrm{inv}} < \frac{\gamma^2 (1 - \mathcal{S}^*/\mathcal{R})}{\mathcal{R}_0^2 (\mathcal{S}^*/\mathcal{R})} \mathcal{S}^* f_P\right]. \tag{S109}$$

So, our analysis of mutant-parent coexistence has an easily-interpreted meaning: a mutant strain will coexist with its parent when its (positive) invasion fitness is below

$$s_{\mathrm{coex}}(f_P) = \frac{\gamma^2 (1 - \mathcal{S}^*/\mathcal{R})}{\mathcal{R}_0^2 (\mathcal{S}^*/\mathcal{R})} \mathcal{S}^* f_P. \tag{S110}$$

We can calculate this probability by treating s_{inv} and f_P as independent random variables, accounting for the probability the mutant survives genetic drift. In this case, their probability densities scale as

$$\rho(s_{\mathrm{inv}}) \sim s_{\mathrm{inv}} \theta(s_{\mathrm{inv}}) e^{-(s_{\mathrm{inv}} - \Delta X)^2 / 2\sigma_{\mathrm{inv}}^2}, \tag{S111}$$

$$\rho(f_P) \sim f_P \theta(f_P) e^{-(f_P + D\lambda)^2 / 2D^2}, \tag{S112}$$

where $D \equiv -2/\mathcal{S}I'(\lambda)$. Finally, $\mathbb{P}_{\text{coex}}$ can be calculated using standard analytic integration methods, giving the results quoted in the main text after applying similar scaling analysis to the previous section. We can also fix either s_{inv} or f_P to calculate a conditional coexistence probability. For example, plugging in a "typical" $f_P = 1/\mathcal{S}^*$ indicates that $\bar{s}_{\text{coex}} = \frac{\gamma^2(1-\mathcal{S}^*/\mathcal{R})}{\mathcal{R}_0^2(\mathcal{S}^*/\mathcal{R})}$ is the typical value of s_{inv} above which the coexistence probability begins to fall.

3.5 Self-Consistency of Replica Approximations

Now that we have expressed the results of our theory in terms of physically interpretable parameters, we can use them to check when the assumptions underlying our analytic approximations are valid. The central assumption we made in Supplementary Note 1.5 was that the resource availabilities h_i are not too far from 1, which should fail at some point in the vicinity of $|g_i| = |1 - h_i| \lesssim 1$. We calculated the typical spread of g_i in terms of σ_{inv} in Supplementary Note 3.2, and found that

$$\text{Std}\,(g_i) \sim (1 - \mathcal{S}^*/\mathcal{R}) \left[\frac{1 - \mathcal{R}_0/\mathcal{R}}{(\mathcal{S}^*/\mathcal{R}) \cdot \mathcal{R}_0} + \sigma_K^2 \right]^{1/2}, \tag{S113}$$

up to an $\mathcal{O}(1)$ factor dependent on $\mathcal{S}^*/\mathcal{S}$. The first term in the brackets should be small over a wide range of parameter values, since $\mathcal{R}_0 \gg 1$. Notably, however, this term causes the $|g_i| \lesssim 1$ approximation to break down when the niche saturation is too small, around

$$\frac{\mathcal{S}^*}{\mathcal{R}} \lesssim \frac{1}{\mathcal{R}_0}. \tag{S114}$$

When this inequality is approached or satisfied, there will not be enough species in the community to cover the space of available resources ($\mathcal{R}_0\mathcal{S}^* \lesssim \mathcal{R}$), so the availability of each resource will be determined by $\mathcal{O}(1)$ rather than many species. Our simulations provide a test of how approaching this bound affects the numerical accuracy of our results: for example, the leftmost points in Fig. 3B (corresponding to $\mathcal{R}_0\mathcal{S}^* = 4\mathcal{R}$) show that the mutant-parent coexistence probability increases more slowly than expected as $\mathcal{S}^*/\mathcal{R}$ declines. Similar quantitative deviations from theory occur in more saturated communities when $\mathcal{R}_0$ is relatively small (e.g. the open circles in Fig. 2D), consistent with our scaling prediction. However, these deviations do not affect our qualitative conclusion that mutant-parent coexistence declines weakly with niche saturation over a wide range of community sizes. Rather, they indicate that in communities where each resource is exploited by only a small number of species, alternative theoretical approaches should be employed.

Self-consistency also requires $\sigma_K \lesssim 1$, so that the spread in resource supply is not too large. We find that our results hold reasonably well even for $\sigma_K = 1$ (Supplementary Fig. 7B-C). However, different approximations must be employed when the abiotic environment has extremely large asymmetries in resource supply. One such example could be some models of crossfeeding, where an $\mathcal{O}(1)$ number of externally supplied metabolites result in the production of many other resources at much lower rates.

In our model, a consequence of small $|g_i|$ is that the total uptake budgets of surviving species have a maximum spread, $\mathrm{Std}\,(X_\mu | f_\mu > 0) \lesssim 1$, which justified the Taylor expansion in Eq. (S24). In other words, species whose uptake budget differences would drive large changes in their relative abundance over a single generation are unlikely to coexist in the community steady-state. Although this condition places bounds on the spread of X_μ among surviving species, it does not imply that the dynamics are effectively neutral (as in Ref. 6). True neutrality requires that fitness differences are small compared to inverse population sizes and inverse equilibration times; because we focus on large populations and long times, there is a large gap in scales between $X_\mu \lesssim 1$ and the emergence of effective neutrality.

The existence of this gap implies that even modest differences in the total uptake budget can still have a significant impact on community structure. Recall that in the simplest version of our model, the uptake budgets of sampled species are drawn from a normal distribution with standard deviation $\mathrm{Std}(X_\mu) = \epsilon/\mathcal{R}_0$. The total scaled fitness variation V_{tot} among sampled species comes from this variance in uptake budget along with resource consumption strategies. If the ratio $\epsilon^2/V_{\mathrm{tot}} \approx 1$, the fates of species will be strongly dependent on their uptake budgets; if $\epsilon^2/V_{\mathrm{tot}} \ll 1$, species fates will mostly depend on their specific resource consumption strategies. From the replica-theoretic results in Supplementary Note 3.2, for uniform resource supply, we have

$$\frac{\epsilon^2}{V_{\mathrm{tot}}} \approx 1 - \frac{\mathcal{S}}{\mathcal{R}} I(\lambda), \tag{S115}$$

$$\approx 1 - \frac{2}{\lambda^2}\frac{\mathcal{S}^*}{\mathcal{R}} \text{ when } \lambda \gg 1. \tag{S116}$$

So, when enough species are sampled (large λ), total uptake budget will be an important determinant of species fate for any fixed value of $\mathcal{S}^*/\mathcal{R}$. Note that if the spread in pure fitness among sampled species is taken to be very small ($\epsilon \ll 1$), then differences in total uptake budget are negligible until enough species have been added to the community to saturate it ($\mathcal{S}^* \approx \mathcal{R}$), as in the top curve in Fig. 2B (1, 10). However, our replica-theoretic assumptions continue to hold for $\epsilon > 1$, because *surviving* species have a narrower spread of X_μ than *sampled* species:

$$\mathrm{Std}\,(X_\mu | f_\mu > 0) \approx \frac{\epsilon}{\mathcal{R}_0}\sqrt{\frac{\int_\lambda^\infty z^2 \mathcal{D}z}{\int_\lambda^\infty \mathcal{D}z} - \left(\frac{\int_\lambda^\infty z\mathcal{D}z}{\int_\lambda^\infty \mathcal{D}z}\right)^2}, \tag{S117}$$

$$\approx \frac{1 - \mathcal{S}^*/\mathcal{R}}{\mathcal{S}^*/\mathcal{R}} \cdot \frac{1 - \mathcal{R}_0/\mathcal{R}}{\mathcal{R}_0} \text{ when } \lambda \gg 1, \tag{S118}$$

for uniform resource supply, where $\mathcal{D}z \equiv (2\pi)^{-1/2} e^{-z^2/2} dz$ is the standard Gaussian integration element. As expected, the spread in fitnesses becomes large when the spread in the g_i's does, around $\mathcal{S}^*/\mathcal{R} \lesssim 1/\mathcal{R}_0$, at which point the replica theory fails due to the number of species alive being too small for a "large-ecosystem" approximation.

Supplementary Note 4: Extensions of Model Assumptions

In this section, we test whether our qualitative conclusions strongly depend on the underlying assumptions of our model, such as the distribution of resource supplies and the method of species sampling. We find that many such changes can be captured by straightforward extensions of our theory, or simply by using the result from the binary consumer resource model directly.

4.1 Non-Uniform Resource Supply

First, let's consider non-uniform resource supply (nonzero σ_K^2), as in (10). If each resource has a supply $\kappa_i = 1 + \delta\kappa_i$, then the availability h_i of that resource will have a component which scales with $\delta\kappa_i$ in addition to a community-dependent component. Thus, mutations that knock out a highly-supplied resource will be less beneficial on average. We can determine the strength of this effect by starting with the integrals over the g_i^a in the second line of Eq. (S49), where the resource supply enters through $F_i(g_i) \equiv \sum_i \kappa_i(g_i + g_i^2/2)$. This portion of the partition function was labeled A_i by Ref. (10) (see their Appendix 2, section 1.3). We can follow their calculation to obtain

$$A_i = \int \frac{d\omega}{2\pi} e^{-\omega^2/2} \left\{ \int_{-\infty}^{1} dg_i \exp\beta \left[-\left(\frac{1}{2x} + \frac{\delta\kappa_i}{2}\right) g_i^2 - \left(\frac{\omega b}{\sqrt{R}} + \delta\kappa_i\right) g_i \right] \right\}^n, \tag{S119}$$

where b is a term independent of $\delta\kappa_i$ whose value does not concern us here. The contents of the brackets are a Gaussian integral over the g_i, centered where the integrand attains its maximum value. Taking non-uniform resource supply, $\delta\kappa_i \neq 0$, will shift this saddle-point value of g_i by approximately $x\delta\kappa_i \ll 1$. The saddle-point value of x was calculated in Ref. (1) as $x = 1 - \mathcal{S}^*/\mathcal{R}$, following our typical substitution of $(\mathcal{S}^*/\mathcal{R})(\mathcal{S}/\mathcal{S}^*)$ for $\mathcal{S}/\mathcal{R}$. This shift in the central value of g_i will shift the invasion fitness of a knock-out mutation targeting that resource by a corresponding amount, since the invasion fitness of a knock-out mutation is simply $g_i/\mathcal{R}_0$. Thus, we can write

$$s_{\text{inv}} = \Delta X - \frac{\delta\kappa_i}{\mathcal{R}_0}\left(1 - \frac{\mathcal{S}^*}{\mathcal{R}}\right) + \mathcal{Z}\sigma_{\text{inv}}(\delta\kappa_i = 0). \tag{S120}$$

for a mutation knocking out resource i. The first term represents the mutation's change in pure fitness, the second term reflects that environmental supply makes some mutations more or less beneficial on average, while the stochastic third term depends on the specific draws of species in the community, as we calculated earlier. We can quantify the relative importance of environment and community by considering the invasion fitness of a mutation in a monoculture community, where invasion fitness *only* depends on the environment:

$$s_{\text{inv}}^{\text{mono}} \approx \Delta X - \frac{\delta\kappa_i}{\mathcal{R}_0}. \tag{S121}$$

The correlation between the invasion fitness of a mutant in monoculture and in the community context illustrates how much the impact of environmental supply is felt through

the "shielding" effects of the community: in the oversampled limit ($\lambda \to \infty$), we find that

$$\rho(s_{\text{inv}}^{\text{comm}}, s_{\text{inv}}^{\text{mono}}) \approx \left(1 + \frac{\mathcal{R} - \mathcal{R}_0}{\mathcal{S}^* \mathcal{R}_0 \sigma_K^2}\right)^{-1/2}, \tag{S122}$$

assuming that $\delta\kappa_i$ are drawn from a distribution with mean zero and variance σ_K^2. In general, non-uniform resource supply will increase the variance in invasion fitness σ_{inv}:

$$\sigma_{\text{inv}} \approx \begin{cases} \gamma\left(1 - \frac{\mathcal{S}^*}{\mathcal{R}}\right)\sqrt{\frac{\mathcal{R}-\mathcal{R}_0}{\mathcal{S}^*\mathcal{R}_0^3} + \frac{\sigma_K^2}{\mathcal{R}_0^2}} & \text{when } \lambda \to -\infty, \\ \gamma\sqrt{2}\left(1 - \frac{\mathcal{S}^*}{\mathcal{R}}\right)\sqrt{\frac{\mathcal{R}-\mathcal{R}_0}{\mathcal{S}^*\mathcal{R}_0^3} + \frac{\sigma_K^2}{2\mathcal{R}_0^2}} & \text{when } \lambda \to \infty. \end{cases} \tag{S123}$$

Note that σ_{inv} still scales with an overall factor of $1 - \mathcal{S}^*/\mathcal{R}$, leaving our previous qualitative results unaffected. Increasing resource supply variation increases σ_{inv} and thus lowers the coexistence probability, by making it less likely that the invasion fitness of an arbitrary beneficial mutation will be small enough to make coexistence possible. Although the theoretical approach assumes $\sigma_K^2 \ll 1$, its quantitative predictions hold reasonably well even when $\sigma_K^2 \sim 1$, as in the case of exponentially distributed K_i (Supplementary Fig. 7B). However, when σ_K^2 increases enough that $\sigma_{\text{inv}} \sim 1/\mathcal{R}_0$, the distribution of fitness effects will be somewhat distorted from the Gaussian prediction (Supplementary Fig. 7C). This is because resource availabilities h_i are non-negative and can only be approximated by a Gaussian when $\text{Std}(h_i) \ll \langle h_i \rangle = 1$.

4.2 Variation in Number of Metabolized Resources

In the binary resource model so far, we have assumed that each resource has an independent probability $\mathcal{R}_0/\mathcal{R}$ of being used by each species. This assumption results in each species using a binomially distributed number of resources, which is tightly peaked when $\mathcal{R}_0 \gg 1$. However, there is no reason to assume that coexisting species cannot differ in their levels of specialism, such that some use many resources and some few. First, it is useful to consider what happens if the parent species uses $\mathcal{R}_P$ resources on average, rather than the $\mathcal{R}_0$ resources that most species in the community use. This extension helps us disentangle what terms in our expressions depend on the typical metabolic overlap between members of the community (encoded by $\mathcal{R}_0$), and what depends on the specific number of resources used by the parent and mutant species. It can also help determine whether generalist or specialist species are more likely to diversify in a given community.

The more resources a given organism uses, the more its growth rate and overall fitness are averaged out between different independent variables, effectively reducing the variance in its abundance as well as the invasion fitness of any particular mutation. So, if $\mathcal{R}_P$ is larger, the parent species is less likely to have particularly high abundance, but the invasion fitness of a strategy mutation it obtains is likely to be smaller in magnitude. The first effect should lower the coexistence probability, while the second should raise it. Performing the

calculation up to our expression for the joint probability of Δ_P and Δ_M gives us

$$A \approx \mathcal{R}_0 \frac{\mathcal{R}_0}{\mathcal{R}_P} \left(1 - \frac{\mathcal{R}_P}{\mathcal{R}}\right), \tag{S124}$$

$$B \approx \frac{\mathcal{R}_0}{\mathcal{R}_P} \frac{\gamma}{2} \left[\frac{\mathcal{R}_0}{\mathcal{R}_P} \left(1 - \frac{\gamma}{2}\right) - \frac{\mathcal{R}_0}{\mathcal{R}}\right], \tag{S125}$$

$$C \approx \frac{\mathcal{R}_0^2}{\mathcal{R}_P^2} \frac{\gamma^2}{4}, \tag{S126}$$

$$D \approx \omega_2 V_P^{1/2} + \lambda V_{\text{tot}}^{1/2}, \tag{S127}$$

$$E \approx \mathcal{R}_0 \frac{\Delta X}{2} + \omega_1 \frac{\mathcal{R}_0}{\mathcal{R}_P} \frac{\gamma}{2} \sqrt{\frac{q}{\mathcal{R}}}. \tag{S128}$$

Here, we have defined $V_P = \frac{\mathcal{R}_0^2(1-\mathcal{R}_P/\mathcal{R})}{\mathcal{R}\mathcal{R}_P} q + \epsilon^2$, reflecting the different variation in overall fitness between the parent and other species in the community. We have once again defined γ such that $\gamma \approx 1$ for a knock-out mutation.

Since most of the community is unaffected by changing $\mathcal{R}_P$, we use the same saddle-point results we calculated before. Thus, the value of σ_{inv} is unchanged, although the invasion fitness of the mutant has a prefactor of $\mathcal{R}_0/\mathcal{R}_P$ to account for the altered number of resources. The inequality we obtain that predicts mutant-parent coexistence (for $\Delta X = 0$) is now given by

$$\mathbb{P}_{\text{P,M live}} \approx \mathbb{P}\left[\mathcal{Z}_1 < \frac{\gamma V_P^{1/2}}{\mathcal{R}_0(1 - \mathcal{R}_P/R)\sqrt{q/R}} \left(\mathcal{Z}_2 - \lambda \frac{V_{\text{tot}}^{1/2}}{V_P^{1/2}}\right)\right], \tag{S129}$$

where $\mathcal{Z}_1$ is once again proportional to the invasion fitness of the mutant, and the $\mathcal{Z}_2$-dependent factor on the RHS is proportional to the abundance of the parent strain. The calculations otherwise proceed as before, yielding the results shown in Supplementary Figure 9A. For these parameter values, it appears that mutant-parent coexistence is more likely for mutations on a background strain which metabolizes many resources, since these mutations tend to have small invasion fitness.

Next, we consider what happens when the number of resources $\mathcal{R}_0$ utilized by *every* species is more broadly distributed, to allow coexistence of generalists and specialists. Specifically, we consider $\mathcal{R}_0 \sim \text{Uniform}(20, 130)$ with $\mathcal{R} = 150$. The actual number of resources used by surviving species will not be uniformly distributed because specialists are more likely to have particularly "lucky" resource strategies; similarly, the typical number of resources used by species which produce a successful knock-out mutant will also be different (Supplementary Fig. 9C). Rather than analytically calculating all of these distributions for this somewhat artificial example, we can use the observed distribution of $\mathcal{R}_0$ and $\mathcal{R}_P$ as empirical inputs to our analytic predictions of fitness effects and mutant-parent coexistence (in particular, plugging in the mean value of $\mathcal{R}_0$, and integrating over the probability distribution of $\mathcal{R}_P$). Doing so yields reasonable predictions for the mutant-parent coexistence probability and distribution of fitness effects (Supplementary Fig. 9B,D), confirming that our results do not critically depend on each member of the community utilizing a similar number of resources.

4.3 Metabolic Trade-offs in Sampling

In our model, we have posited a soft metabolic trade-off where each sampled organism μ has an identically distributed "uptake budget" e^{X_μ} which must be divided between the resources it consumes; thus, organisms which consume more resources are generally less effective at consuming any individual one. Other work has considered different forms of such trade-offs (10, 11) or none at all, which can alter the number of species that coexist in the community (3). For example, Refs. (1) and (10) also consider a soft metabolic trade-off, but where the cost of consuming additional resources is associated with a larger average death rate rather than with taking a portion of a fixed overall budget. While these models describe very similar final communities, the main difference is that the death rate trade-off model predicts that as sampling depth $\mathcal{S}/\mathcal{S}^*$ increases, the fitness effect of using an additional resource becomes slightly deleterious on average, such that the mean invasion fitness of knock-out mutations is small but positive (Supplementary Fig. 8A). Accounting for this small fitness effect through an effective change in pure fitness ΔX in knock-out and knock-in mutants brings the mutant-parent coexistence probabilities in agreement between the models (Supplementary Fig. 8B).

Another alternative is to eliminate metabolic trade-offs in sampling entirely (3). In terms of the model we have discussed so far, a lack of trade-offs effectively grants organisms which use more resources a larger budget to divide among those resources. This change causes organisms which use more resources to be overrepresented in the population due to their higher effective fitness, and lowers the niche saturation $\mathcal{S}^*/\mathcal{R}$ of resultant communities. However, when these empirical changes in effective $\mathcal{R}_0$ and $\mathcal{S}^*/\mathcal{R}$ are accounted for, the distribution of fitness effects and mutant-parent coexistence probability are well-described by the original model with trade-offs (Supplementary Fig. 8C-D). It is important to distinguish metabolic trade-offs in *sampled* species from trade-offs in mutations, which can be captured by changing the value of ΔX as described in the main text. These trade-offs may be quite different over short evolutionary timescales: for example, first-step mutations might introduce metabolic inefficiencies which are reduced by subsequent mutations.

4.4 Continuous Resource Usage

We have generally considered a binary model of resource use, where a natural parameter to describe metabolic overlap is the number of resources $\mathcal{R}_0$ used by a typical species. A simple continuous analogue to binary resource usage is drawing resource strategies from a Dirichlet distribution, $\boldsymbol{\alpha}_\mu \sim \mathrm{Dirichlet}(\mathcal{R}_0/\mathcal{R}, 1)$. Typical resource consumption vectors sampled from this distribution are qualitatively similar to binary ones; in particular, they will have $\sim \mathcal{R}_0$ entries which are $\mathcal{O}(\mathcal{R}_0^{-1})$, with the remaining entries much smaller. As an analogue to knock-out mutations, we chose a random entry of the parent organism's strategy vector with value at least $\gamma/\mathcal{R}_0$, and reduced it by $\gamma/\mathcal{R}_0$, where $\gamma = 1$ represents a full knock-out. We also considered "global-effect" mutations which slightly perturb each element of the resource use vector. Specifically, a global strategy mutation $\Delta\boldsymbol{\alpha}$ was constructed by multiplying each element of $\boldsymbol{\alpha}$ by an independent Gaussian random variable with mean 0 and variance $1/\mathcal{R}_0$, then by rescaling such that $\sum_i \Delta\alpha_i = 0$ and $|\Delta\boldsymbol{\alpha}| = \gamma/\mathcal{R}_0$.

Using resource consumption vectors drawn from a continuous distribution in this manner left the quantitative properties of our communities relatively unchanged (Supplementary Fig. 3A-B, Supplementary Fig. 5).

As a more extreme example of continuous resource usage, we can consider a community where all resources are significantly utilized by all species, but at slightly different levels. For example, we can draw $\boldsymbol{\alpha}_\mu \sim \text{Dirichlet}(B, 1)$ for $B \gg 1$. In this case, the distribution of resource consumption strategies will approach a Gaussian, $\alpha_{\mu,i} \sim \mathcal{N}(\mathcal{R}^{-1}, \mathcal{R}^{-1}/\sqrt{B})$; similar sampling schemes have been analyzed before, as in (3). With these Gaussian resource consumption strategies, the number of resources $\mathcal{R}_0$ utilized by a typical species no longer has intuitive meaning. However, we can generalize our conclusions from binary resource usage by defining an effective $\mathcal{R}_0$ such that the binary model and the Gaussian model predict the same level of variance $\|\Delta\boldsymbol{\alpha}_{\text{comm}}\|$ in resource consumption strategies:

$$\mathcal{R}_0^{\text{eff}} \approx \mathcal{R} - \mathcal{R}/B. \tag{S130}$$

Thus, a model where organisms use all resources at slightly different levels is equivalent to a binary resource usage model where organisms use *almost* all resources, in terms of the metabolic overlap between species. We can define mutations in this framework by perturbing the parent's resource usage $\boldsymbol{\alpha}_P$ toward a different, i.i.d. resource usage profile $\boldsymbol{\alpha}'$:

$$\alpha_{M,i} = \mathcal{R}^{-1} + \sqrt{1-\Gamma}\left(\alpha_{P,i} - \mathcal{R}^{-1}\right) + \sqrt{\Gamma}\left(\alpha'_i - \mathcal{R}^{-1}\right), \tag{S131}$$

where $\Gamma \leq 1$ is a mutation effect size parameter describing how much of the initial resource use profile $\boldsymbol{\alpha}_P$ is retained.

What should the parent-mutant coexistence probability be for such a mutation? Plugging in the $\|\Delta\boldsymbol{\alpha}\|$ and $\|\Delta\boldsymbol{\alpha}_{\text{comm}}\|$ for this assembly process into Eq. (9), we find

$$\mathbb{P}_{\text{coex}} \approx \frac{\Gamma}{\mathcal{S}^*/\mathcal{R}} \tag{S132}$$

for $\Gamma \ll 1$. Indeed, we find that the mutant-parent coexistence probability for this community is well-predicted by naively using the binary resource predictions with the value of $\mathcal{R}_0$ defined in Eq. (S130), and has the expected qualitative dependence on Γ and B (Supplementary Fig. 3C).

4.5 Specialist Community Assembly

As discussed in Supplementary Note 3.5, the replica-theoretic approach involves a "large-ecosystem" approximation which is only valid when $\mathcal{S}^*\mathcal{R}_0 > \mathcal{R}$, such that each resource is consumed by many species. Species coexisting in such ecosystems tend to not have large differences in their uptake budgets X_μ, although these differences can still be ecologically relevant. Here, we consider a simple "specialist assembly" procedure, as a representative example that violates this condition. Imagine that an initial community contains $\mathcal{S}^*$ species, each of which use exactly one resource with no overlap. These species will trivially coexist, at abundances which match the supply of their private resource, $n_\mu = K_\mu$.

Then, suppose that one organism P acquires a partial knock-in mutation which enables it to use another resource ν. We label the mutant M, so that

$$\alpha_{P,\mu} = 1 \qquad \alpha_{P,\nu} = 0, \tag{S133}$$

$$\alpha_{M,\mu} = 1 - \gamma_{\text{spec}}, \qquad \alpha_{M,\nu} = \gamma_{\text{spec}}, \tag{S134}$$

where γ_{spec} describes the effect size of the mutation. While this ecosystem is clearly a caricature, it is a useful example far from the regime where our results should be applicable with which to compare our qualitative results.

What is the impact of the mutation? If no other organism utilizes resource ν, then the mutant will trivially always survive and coexist with its parent. However, if another organism O is already utilizing resource ν, then the mutant will survive if its fitness is greater, $X_M > X_O$. If the mutant survives, it will either replace its parent strain, or coexist with it while replacing O. Specifically, mutant-parent coexistence occurs if the following condition is met:

$$\mathbb{P}_{\text{coex}} = \mathbb{P}\left[\frac{K_\mu}{K_\mu + K_\nu} < \gamma_{\text{spec}}\right], \tag{S135}$$

as well as the trivial case where no other organism utilizes ν. Although the condition for coexistence is different from the more complex assembly procedure we discuss elsewhere, we still find that it is nonzero even in fully saturated communities (Supplementary Fig. 3D). Thus, while our results do not directly extend to these communities, their existence does not invalidate our main conclusions. Furthermore, we expect that the sparser interaction networks of these communities makes them more amenable to previous analysis focusing on small numbers of species and resources (4).

Supplementary Note 5: Simulations and Numerics

5.1 Community Simulations

Throughout this work, we have performed numerical simulations of community assembly in order to verify and extend our analytical results. These simulations were performed by randomly sampling species and solving the optimization problem corresponding to ecological equilibrium (described in Supplementary Note 1.5), rather than explicitly solving the abundance dynamics: the final outputs are the resource availabilities h_i and the surpluses Δ_μ. The code for these simulations is based on that in Ref. (10), and can be found (with example outputs) on Zenodo (14).

Much of our analysis relies on identifying whether individual species are present in the population or extinct. Mathematically, these cases correspond to whether Δ_μ is zero or negative. Numerically, however, Δ_μ is never precisely zero, necessitating the definition of an "extinction threshold." Any species with a surplus Δ_μ below the extinction threshold, a small negative number, is classified as extinct. We typically defined the extinction threshold as $-10^{-3} \cdot \mathrm{Std}(h_i)/\mathcal{R}_0$, several orders of magnitude smaller than the typical fitness effect of a knock-in or knock-out mutation.

In Supplementary Figure 10, we demonstrate that this is a reasonable choice of threshold. In order to be numerically sensible, there should be a clear separation between typical values of Δ_μ for surviving and extinct species, and the threshold should be well within this gap. We verified this by plotting the number of putative surviving species as a function of the extinction threshold. In the neighborhood of the threshold we have chosen, the number of putative survivors is independent of the threshold, suggesting that there is a clear separation between alive and extinct species and that our choice of threshold should not heavily impact our results. We also analyzed the values Δ_μ for each strain before and after mutant invasion in order to ensure that we were reliably identifying species that go extinct. A scatterplot of initial vs. final Δ_μ values shows that almost all species are clearly separated into a "surviving" cluster and an "extinct" cluster, again suggesting that our results are not due to numerical artifacts.

5.2 Theory Numerics

While the main text and the other Supplementary Notes describe the novel theory key to our work, some theory curves shown in figures involve minor post-processing steps or slightly more precise calculations than those quoted in the text. For convenience, we summarize these steps (which are also performed in our code supplement) below.

Distribution of fitness effects (e.g. Fig. 2C-D): Our theoretical results predict that the distribution of fitness effects (DFE) follows a Gaussian distribution with mean zero and standard deviation σ_{inv}, which is calculated by plugging our parameters into the equations in Supplementary Note 3.2. The mean of this distribution is taken as a variable ΔX (sometimes assumed to be zero).

Coexistence probability (e.g. Fig. 3): We calculate $\mathbb{P}_{\text{coex}}$ by analytically integrating Eq. (S107) over the probability distributions of s_{inv} and $\mathcal{Z}_2$. Both distributions are truncated Gaussians multiplied by a linear term: $p(s) \sim s\rho(s)\theta(s)$, where $\rho(s)$ is the distribution of fitness effects above and $\theta(s)$ is the Heaviside function. $\mathcal{Z}_2$ is related to the parent relative abundance via Eq. (S101), and thus takes on a similar distribution: $p(z_2) \sim (z_2 - \lambda)e^{-z_2^2/2}\theta(z_2 - \lambda)$. In the inset of Fig. 3D, $\mathbb{P}_{\text{coex}}$ is conditioned on s_{inv}, so it is not integrated over. In Supplementary Fig. 6, we condition on both s_{inv} and $\mathcal{Z}_2$ (via f_P), so Eq. (S107) determines a line in the s_{inv}-f_P plane above which mutant-parent coexistence is permitted.

Relative abundance (e.g. Fig. 4D): Relative abundance f_μ is calculated from simulation output by identifying surviving species using the resource surplus Δ_μ (as described in Supplementary Note 5.1) and then using a non-negative least-squares solver to invert the relationship $h_i^{-1} = (\mathcal{R}/\kappa_i)\sum_\mu f_{\mu\in\text{Survivors}}\alpha_{\mu,i}e^{X_\mu}$.

Simluating model extensions (e.g. Supplementary Fig. 3C): For some extensions of the model, the theoretical expectation of $\mathcal{S}^*/\mathcal{R}$ was not explicitly calculated. Rather than choosing parameters to target a specific value of $\mathcal{S}^*/\mathcal{R}$, simulations were run with a fixed value of $\mathrm{Std}(X_\mu)$ and varying log-spaced $\mathcal{S}/\mathcal{R}$ to cover a range of $\mathcal{S}^*/\mathcal{R}$. To make smooth theoretical predictions from this data, the empirical values of $\lambda(\mathcal{S}^*/\mathcal{R})$ were linearly interpolated

over the sampled data. In Supplementary Fig. 8C-D, the sampled $\mathcal{R}_0$ differed from the $\mathcal{R}_0$ among survivors due to the systematic advantage in using more resources; as such, the empirical average $\mathcal{R}_0$ among surviving species was used as input to the theory.

Figure 2B: We numerically solve the system of equations in Supplementary Note 3.2 for λ and find $\mathcal{S}^*/\mathcal{R}$ by inverting Eq. (S88).
Figure 2E: We calculate the correlation between the expressions in Equations (S120) and (S121) in terms of σ_{inv}.
Supplementary Figure 1: The set of equations in Eq. (S1a) were numerically solved using a standard MATLAB differential equation solver, with random initial conditions corresponding to low species abundances and resource concentrations at their steady-state in the absence of species.
Supplementary Figure 8A-B: An effective $\Delta X = m/\mathcal{R}$ is determined by Eq. (S89) with $\chi = 0$.
Supplementary Figure 9A: In the left panel, $\mathbb{P}_{\text{coex}}$ is determined by integrating Eq. (S129) over its random variables rather than Eq. (S107). $\mathcal{Z}_1$ is a standard Gaussian truncated to take positive values, while $\mathcal{Z}_2$ is a standard Gaussian truncated to take values above $\lambda\sqrt{V_{\text{tot}}/V_P}$.
Supplementary Figure 9B-D: In addition to the changes discussed in "Simulating model extensions" above, the mean values of $\mathcal{R}_0$ and $\mathcal{R}_P$ among surviving species were linearly interpolated over the sampling regions of each condition to produce the theory curves in panel B. While the mean value of $\mathcal{R}_0$ was used for the DFE in panel D, the entire empirical distribution of $\mathcal{R}_P$ (i.e., the number of resources used by species which were randomly drawn to produce mutants across all simulation trials) was numerically integrated over to account for the tail effects.

References

[1] Tikhonov, M. & Monasson, R. Collective Phase in Resource Competition in a Highly Diverse Ecosystem. Phys. Rev. Lett. **118**, 048103 (2017).

[2] Advani, M., Bunin, G. & Mehta, P. Statistical physics of community ecology: A cavity solution to MacArthur's consumer resource model. J. Stat. Mech., 033406 (2018).

[3] Cui, W., Marsland, R. III & Mehta, P. Effect of Resource Dynamics on Species Packing in Diverse Ecosystems. Phys. Rev. Lett. **125**, 048101 (2020).

[4] Good, B., Martis, S. & Hallatschek, O. Adaptation limits ecological diversification and promotes ecological tinkering during the competition for substitutable resources. Proc. Natl. Acad. Sci. U.S.A. **115**, E10407-E10416 (2018).

[5] Amicone, M. & Gordo, I. Molecular signatures of resource competition: Clonal interference favors ecological diversification and can lead to incipient speciation. Evol. **75**, 2641-2657 (2021).

[6] Posfai, A., Taillefumier, T. & Wingreen, N. S. Metabolic Trade-Offs Promote Diversity in a Model Ecosystem. Phys. Rev. Lett. **118**, 028103 (2017).

[7] MacArthur, R. Species Packing, and What Competition Minimizes. Proc. Natl. Acad. Sci. U.S.A. **64**, 1369-1371 (1969).

[8] Liu, Y., Hu, J., Lee, H. & Gore, J. Complex ecosystems lose stability when resource consumption is out of niche. Preprint at biorxiv.org/content/10.1101/2023.11.30.569477v1 (2023).

[9] Mehta, P. & Marsland, R. III. Cross-feeding shapes both competition and cooperation in microbial ecosystems. Preprint at biorxiv.org/content/10.1101/2021.10.10.463852v1 (2021).

[10] Tikhonov, M. & Monasson, R. Innovation rather than improvement: A solvable high-dimensional model highlights the limitations of scalar fitness. J. Stat. Phys. **172**, 74-104 (2018).

[11] Caetano, R., Ispolatov, Y. & Doebeli, M. Evolution of diversity in metabolic strategies. eLife **10**, e67764 (2021).

[12] Doebeli, M. *The Adaptive Landscape in Evolutionary Biology* Ch. 14 (Oxford University Press, Oxford, 2013).

[13] Gillespie, J. *Population Genetics: A Concise Guide* (Johns Hopkins University Press, Baltimore, MD, 2004).

[14] McEnany, J. & Good, B. H. Predicting the First Steps of Evolution in Randomly Assembled Communities – Code and Data Supplement. Zenodo doi.org/10.5281/zenodo.13207464 (2024).
